# ArhGEF37 assists dynamin 2 during clathrin-mediated endocytosis

**DOI:** 10.1242/jcs.226530

**Published:** 2019-05-08

**Authors:** Abhiyan Viplav, Tanumoy Saha, Jan Huertas, Philipp Selenschik, Mirsana P. Ebrahimkutty, David Grill, Julia Lehrich, Andreas Hentschel, Monika Biasizzo, Simone Mengoni, Robert Ahrends, Volker Gerke, Vlad Cojocaru, Jürgen Klingauf, Milos Galic

**Affiliations:** 1 DFG Cluster of Excellence ‘Cells in Motion’, University of Muenster, 48149 Muenster, Germany; 2 Institute of Medical Physics and Biophysics, University of Muenster, 48149 Muenster, Germany; 3 Computational Structural Biology Group, Dept. of Cell and Developmental Biology, Max Planck Institute for Molecular Biomedicine, Muenster, 48149 Muenster, Germany; 4 Institute for Medical Biochemistry, ZMBE, University of Muenster, 48149 Muenster, Germany; 5 Leibniz-Institut für Analytische Wissenschaften, ISAS, 44139 Dortmund, Germany

**Keywords:** BAR domain, Endocytosis, Clathrin-mediated endocytosis, CME, Dynamin 2, ArhGEF37

## Abstract

Clathrin-mediated endocytosis (CME) engages over 30 proteins to secure efficient cargo and membrane uptake. While the function of most core CME components is well established, auxiliary mechanisms crucial for fine-tuning and adaptation remain largely elusive. In this study, we identify ArhGEF37, a currently uncharacterized protein, as a constituent of CME. Structure prediction together with quantitative cellular and biochemical studies present a unique BAR domain and PI(4,5)P_2_-dependent protein–membrane interactions. Functional characterization yields accumulation of ArhGEF37 at dynamin 2-rich late endocytic sites and increased endocytosis rates in the presence of ArhGEF37. Together, these results introduce ArhGEF37 as a regulatory protein involved in endocytosis.

## Abstract (precis)

**Summary:** Accumulation of ArhGEF37 at dynamin 2-rich late endocytic sites yields increased rates of endocytosis.

## INTRODUCTION

Endocytosis is essential to internalize membranes and extracellular material (Hopkins et al., 1985; Rosenbluth and Wissig, 1964). As such, it contributes to a variety of cellular functions during development and in the adult organism, including trans-cellular signaling (Ferguson and De Camilli, 2012; Traub, 2009), nutrient uptake (McMahon and Boucrot, 2011), maintenance of membrane tension (Boulant et al., 2011; Ferguson et al., 2009) and pathogen entry (Saheki and De Camilli, 2012). In mammalian cells, uptake occurs among others through CME, the clathrin-independent pathway or caveolae (Sorkin, 2000), with CME being the principal mechanism (Doherty and McMahon, 2009; Ferguson et al., 2009; Ferguson and De Camilli, 2012; Robinson, 2004).

CME is initiated by invagination of the plasma membrane (PM) through clustering of curvature-inducing proteins (Daumke et al., 2014; Frost et al., 2009; McMahon et al., 2010). Upon indentation, the PM is then further deformed by polygonal clathrin scaffolds (i.e. clathrin-coated pits, CCPs), ultimately creating a spherical invagination (Ferguson and De Camilli, 2012; McMahon and Boucrot, 2011). In a final step, assembly and contraction of dynamin at the neck of CCP, supported by actin polymerization (Almeida-Souza et al., 2018), triggers scission of the newly formed endocytotic vesicle from the membrane (Ferguson and De Camilli, 2012; Morlot et al., 2012; Taylor et al., 2011).

The whole process, which takes on average 35–50 s (Ehrlich et al., 2004; Shamir et al., 2016), is characterized by finely orchestrated recruitment and dissociation of various endocytotic proteins (Posor et al., 2013; Taylor et al., 2011). Intriguingly, roughly one-third of the currently 34 identified endocytotic proteins contain curvature-sensitive domains (Daumke et al., 2014; Qualmann et al., 2011). As endocytosis is associated with dynamic changes in membrane geometry (e.g. low/high curvature; spherical/cylindrical/saddle shape), it has been put forward that the combination of unique curvature-sensing properties (Gallop et al., 2006; Henne et al., 2007; Shimada et al., 2007) and additional functional domains present in these proteins (Qualmann et al., 2011; Taylor et al., 2011) are crucial for the correct spatio-temporal assembly of the endocytotic machinery.

Considering the prevalence of Bin–Amphiphysin–Rvs (BAR) domain-containing proteins during endocytosis (Taylor et al., 2011), and curvature-dependent properties associated with many of its family members (Peter et al., 2004), we set out to identify uncharacterized proteins containing a BAR domain and probe their role in endocytosis.

## RESULTS

**Fig. 1.**
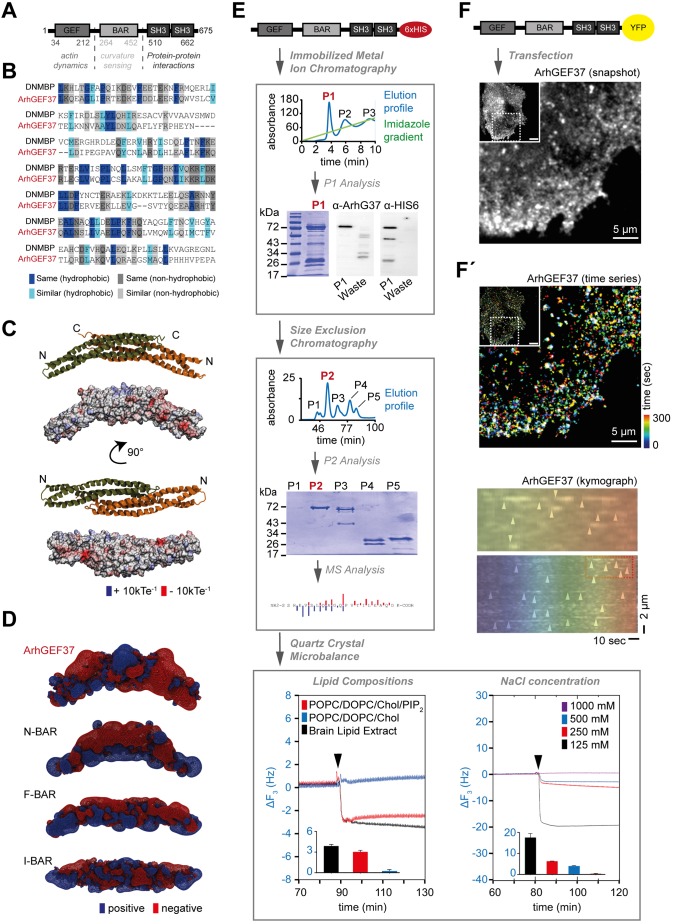
**Structure prediction and recruitment dynamics of ArhGEF37.** (A) Domain organization of ArhGEF37. (B) Sequence alignment of the BAR domains of ArhGEF37 and DNMBP show conserved hydrophobic (blue) and non-hydrophobic (gray) residues. BAR domain alignment shows 24.9% identical (50/201) and 28.4% similar (57/201) amino acids between ArhGEF37 and DNMBP. (C) Antiparallel BAR-domain homodimer of ArhGEF37 as shown in the first and third model (monomers in green and orange); surface colored model (second and fourth model) show the electrostatic potential (red, −10 kTe^−1^; blue, +10 kTe^−1^). (D) Isosurface of BAR-domain dimers of ArhGEF37, amphiphysin (N-BAR, PDB ID: 1URU), FCHO2 (F-BAR, PDB code: 2V0O) and IRSp53 (I-BAR, PDB ID: 1Y2O); red and blue show isosurfaces of −0.75 kTe^−1^ and 0.75 kTe^−1^, respectively. (E) ArhGEF37 binds to planar model membranes. Flow-chart depicting ArhGEF37 purification and membrane-binding assay. Top box: Immobilized metal ion chromatography elution profile showing three peaks (P1, P2, P3), the imidazole elution gradient is indicated in green. Below, Coomassie Blue staining of the P1 fraction (left) and western blot analysis (right) using anti-ArhGEF37 and anti-HIS-tag antibodies as indicated. Middle box: Size-exclusion chromatography (top) followed by Coomassie Blue staining (middle) and mass spectrometry (bottom) establish ArhGEF37 as the main component of the P2 fraction. Bottom box: Membrane binding of purified ArhGEF37 probed using QCM. ArhGEF37 binding to various lipid compositions (left) and at different NaCl concentrations (right). Bar graphs depict mean frequency change (*n*=4 experiments/condition). (F) ArhGEF37 forms puncta at the basal membrane. Main image shows magnification of boxed area of inset (top left) image. (F′) ArhGEF37 puncta are transient. (Top) Time series (main image) showing magnification of boxed area of inset (top left) image. Protein dynamics are depicted in false colors. (Bottom) Kymograph of line scan shows ArhGEF37 kinetics (arrowheads); boxed area in bottom image is shown magnified above. Scale bars: 5 µm (F, F′ top panel), 2 µm (F′ bottom panel). See also Fig. S1 and Movie 1*.*

To identify potential candidates, we performed a database search for uncharacterized BAR proteins (data not shown). This focused bioinformatics screen led us to ArhGEF37, a 676 amino acid-long protein of unknown function. Published expression profiles indicate differential expression across tissues (https://www.proteinatlas.org, http://biogps.org) and age (http://www.brainspan.org/). According to basic local alignment search tools, ArhGEF37 consists of an N-terminal actin-regulatory Rho guanine nucleotide exchange factor (RhoGEF) domain, followed by a BAR domain and two Src homology 3 (SH3) domains associated with protein–protein interaction (Fig. 1A). To gain further insight into protein organization, we examined the secondary structure of individual domains by using PSIPRED (Jones, 1999). We observed α-helixes and coil structures in the GEF and BAR domain, whereas β-strands and coils were predicted for both SH3 domains (Table S1). A subsequent search using pGenTHREADER yielded predominantly CME-associated proteins (Table S2). Specifically, when searching separately for homologs with known structure for each domain, the BAR domain presented the N-BAR domain proteins amphiphysin1 and 2, (PDB IDs: 2FIC, 1URU, 4ATM) as the three homologs with the highest certainty scores, whereas both SH3 domains yielded DNMBP (PDB ID: 1UHC) as the best match (Tables S2 and S3). Likewise, the closest homolog of ArhGEF37 with respect to domain structure (i.e. DNMBP) showed high levels of conserved hydrophobic (blue) and non-hydrophobic residues (gray) for the BAR domain (Fig. 1B and Fig. S1A).

Next, using the homologs listed in Table S2, we built homology models by using MODELLERv9.19 (Eswar et al., 2007, see Materials and Methods) for each domain present in ArhGEF37 (Fig. S1B). To model the BAR domain homo-dimer of ArhGEF37, we symmetrically superimposed two modeled BAR domains analogous to the published crystal structure (PDB ID: 1URU) (Peter et al., 2004). As in the templates used to build the model, we observed an elongated banana-shaped dimer with positive patches (blue) on the convex (i.e. positively curved) surface (Fig. 1C and PDB file). Strikingly, however, isosurface comparisons between ArhGEF37 and prototypic BAR domain members (i.e. N-BAR, F-BAR and I-BAR) yielded stark differences in shape and surface-charge distribution (Fig. 1D).

Individual BAR-domain subtypes substantially differ in their membrane interactions (Peter et al., 2004). To explore possible membrane-binding properties of ArhGEF37, we expressed C-terminal His_6_-tagged ArhGEF37 in *E. coli* and purified the protein by using immobilized metal ion affinity chromatography (Fig. 1E, top). Coomassie Blue staining followed by immuno-staining of ArhGEF37 and HIS_6_ confirmed purification of intact ArhGEF37 (Fig. 1E, top and Fig. S1C). To eliminate unspecific proteins from the sample, we performed size-exclusion chromatography, and probed for protein identity using mass spectroscopy. With 41 unique sequence hits, covering 77% of the whole protein sequence, we confirmed ArhGEF37 to be the principal component of P2 (Fig. 1E, middle). By using purified ArhGEF37, we finally analyzed its binding ability to planar membranes using quartz crystal microbalance (Sauerbrey, 1959). Strikingly, we observed binding of ArhGEF37 to model membranes formed from brain lipids (Fig. 1E, bottom left; Fig. S1D). We further observed binding of ArhGEF37 to model membranes composed of POPC/DOPC/cholesterol/PI(4,5)P_2_ but not to membranes devoid of charged PI(4,5)P_2_, establishing charge-dependent protein-membrane interactions. Consistently, increases in the concentration of NaCl, which reduces electrostatic attraction by shielding charged lipid head groups and amino acids, prevented membrane binding of ArhGEF37 (Fig. 1E, bottom right and Fig. S1E).

Next, we aimed to elucidate the function of ArhGEF37 in a cellular context. For this, cells were transfected with ArhGEF37, and the basal membrane was imaged by using spinning disk confocal microscopy (Fig. 1F). Intriguingly, we observed a punctate pattern of ArhGEF37 that rapidly appeared and disappeared (Fig. 1F′, Movie 1).

**Fig. 2.**
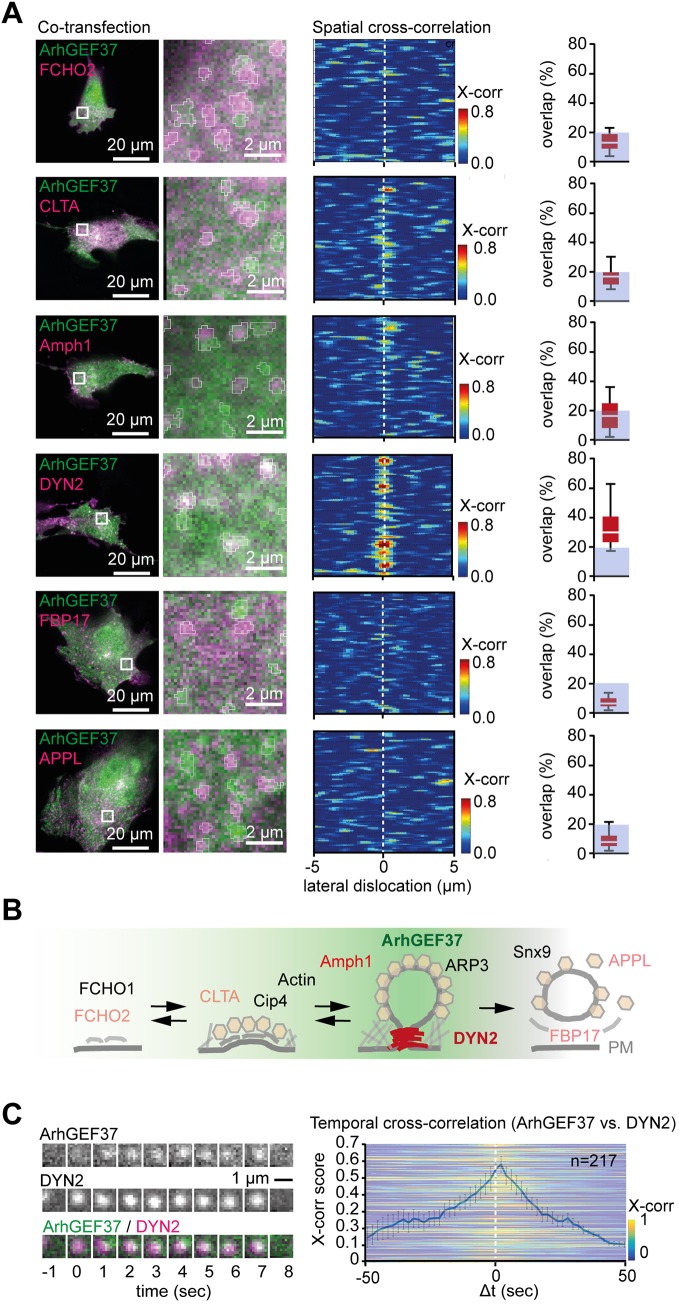
**ArhGEF37 recruitment to late endocytotic sites coincides with DYN2.** (A) ArhGEF37 and DYN2 show high spatial cross-correlation. Left column: Cells coexpressing ArhGEF37 (green) and different endocytotic proteins (magenta). Boxed areas in left images are shown magnified at the right, depicting Wavelet-transformed puncta used for analysis (circumference of Wavelet masks are shown in white). Middle column: Scores of spatial cross-correlation of ArhGEF37 vs endocytotic proteins. Vertical dashed lines at position 0 represents cross-correlation value without lateral pixel shift. Right column: Quantification of overlap percentage for ArhGEF37 vs other endocytotic proteins (FCHO2: 14±8%, *n*=15 cells; CLTA: 16±6%, *n*=16 cells; Amph1: 17±10%, *n*=14 cells; DYN2: 32±11%, *n*=15 cells; FBP17: 9±6%, *n*=13 cells; APPL1: 8±4%, *n*=12 cells). Gray boxes (0-20%) in graphs act as guidance to the eye. (B) Graphical summary of CME, depicting appearance of ArhGEF37 (green) and endocytotic proteins used in this study (red). (C) Temporal cross-correlation of ArhGEF37 vs DYN2. Time-series of ArhGEF37 (green) and DYN2 (magenta) to the left. Next to it, temporal cross-correlation of DYN2 vs ArhGEF37 (*n*=217 ROIs) yields maximal peak at +1, indicative of DYN2 recruitment prior to ArhGEF37. Error bars represent ±s.e.m. Scale bars: 20 µm, 2 µm (A); 1 µm (C). See also Fig. S2.

To further characterize the spatio-temporal kinetics of these puncta, we coexpressed fluorescently labeled full-length ArhGEF37 with several proteins present at different stages of CME (Fig. 2A), with all data first being subjected to *À trous* wavelet filtering (Olivo-Marin, 2002) (Fig. S2A-C and Movie 2). When comparing the overlap percentage of ArhGEF37 with the other proteins (Materials and Methods), we found the highest ArhGEF37 colocalization with dynamin 2 (DYN2), followed by colocalization with clathrin light chain (CLTA) or amphiphysin 1 (Amph1). No apparent colocalization of ArhGEF37 was observed with FCHO2, FBP17 or APPL1 (Fig. 2A, left). To further probe these findings, images were examined by using spatial cross-correlation (Matis et al., 2012). Consistent with results from the overlap analysis, strongest cross-correlation scores were observed for ArhGEF37 and DYN2, followed by CLTA and Amph1. Again, no cross-correlation was apparent for FCHO2, FBP17 or APPL1 (Fig. 2A, middle), suggesting recruitment of ArhGEF37 to coincide predominantly with DYN2 during late stages of CME (Fig. 2B).

To gain further insight into the recruitment dynamics of ArhGEF37, we performed a temporal cross-correlation analysis (Fig. S2D). To establish a baseline, we first took advantage of published results on the recruitment kinetics of key endocytotic components (i.e. CLTA, DYN2 and Snx9) (Fig. S2E, left). When using DYN2 as reference, the maximal peak for CLTA shifted towards the left (i.e. CLTA precedes DYN2), whereas Snx9 moved to the right (i.e. Snx9 follows DYN2) (Fig. S2E, right), establishing accurate temporal analysis. We then probed the recruitment kinetics of ArhGEF37 vs DYN2. Using cells co-transfected with full-length ArhGEF37 and DYN2, we observed a maximal correlation score for ArhGEF37 at +1 s (Fig. 2C), suggesting DYN2 enrichment slightly before ArhGEF37.

**Fig. 3.**
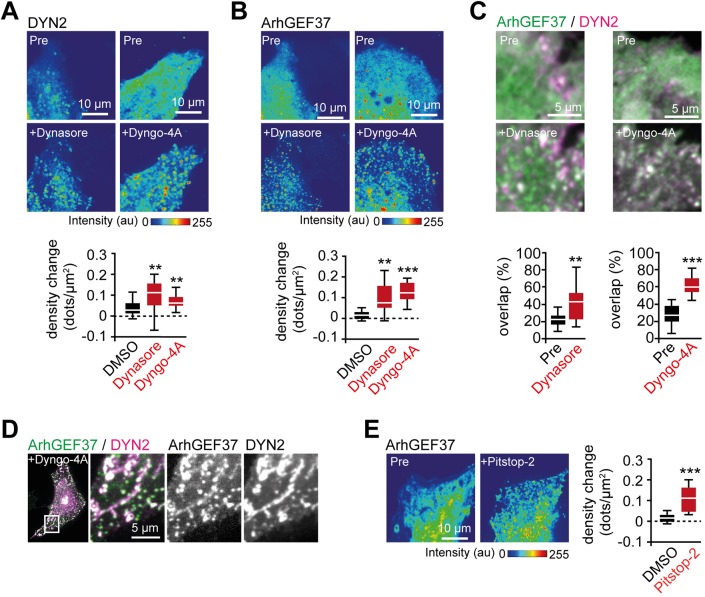
**Endocytosis inhibitors trigger co-enrichment of DYN2 and ArhGEF37.** (A) Dynamin inhibitors trigger DYN2 enrichment. Cells expressing DYN2 before and 30 min after addition of Dynasore (left) and Dyngo-4A (right) (DMSO: 0.04±0.04 dots/µm^2^, *n*=13 cells; Dynasore: 0.1±0.06 dots/µm^2^, *n*=12 cells; Dyngo-4A: 0.07±0.03 dots/µm^2^, *n*=17 cells). (B) Dynamin-inhibitors trigger enrichment of ArhGEF37. Cells expressing ArhGEF37 before and 30 min after incubation with Dynasore (left) and Dyngo-4A (right) (DMSO: 0.01±0.03 dots/µm^2^, *n*=13 cells; Dynasore: 0.09±0.06 dots/µm^2^, *n*=12 cells; Dyngo-4A: 0.09± 0.03 dots/µm^2^, *n*=17 cells). (C) Increased colocalization of ArhGEF37 (green) and DYN2 (magenta) upon addition of Dynasore (pre: 22±8%, black; post: 42± 22%, red; *n*=11 cells) and Dyngo-4A (pre: 27±11%, black; post: 61±10%, red; *n*=13 cells). (D) Addition of Dyngo-4A triggers co-enrichment of ArhGEF37 (green) and DYN2 (magenta) in tubular structures. (E) The clathrin-specific inhibitor Pitstop-2 triggers ArhGEF37 enrichment at the PM. Cells expressing ArhGEF37 before and 30 min after incubation (DMSO: 0.01±0.03 dots/µm^2^, *n*=13 cells; Pitstop-2: 0.08±0.04 dots/µm^2^, *n*=13 cells). Scale bars: 10 µm (A,B,E); 5 µm (C,D). See also Fig. S3 and Movies 2, 3.

To further elucidate the link between ArhGEF37 and DYN2, we probed protein dynamics in the presence of endocytosis inhibitors. Consistent with published work, addition of Dynasore (Macia et al., 2006) and Dyngo-4A (Harper et al., 2011) both led to an increase in DYN2 puncta at the PM (Fig. 3A), whereas no changes were observed for a cytosolic reference protein (Fig. S3A). Similarly, dynamin inhibition increased ArhGEF37 puncta density at the PM (Fig. 3B and Fig. S3B). In addition, we find a significant increase in overlap percentage of DYN2 and ArhGEF37 puncta in the presence of Dynasore and Dyngo-4A (Fig. 3C), as well as colocalization of both proteins at tubular structures that formed at the PM in the presence of Dyngo-4A (Fig. 3D). We further observed an increase in ArhGEF37 puncta upon expression of dominant-negative DYN2 (K44A) (Damke et al., 1994) (Fig. S3C,D), as well as upon addition of the clathrin-specific endocytosis inhibitor Pitstop-2 (von Kleist et al., 2011) (Fig. 3E). Collectively, all perturbation results are consistent with kinetic data showing ArhGEF37 enrichment at late endocytotic sites. Importantly, as changes in temperature yield stark differences in inhibitor efficacy (Fig. S3E), all experiments were performed at 37°C.

**Fig. 4.**
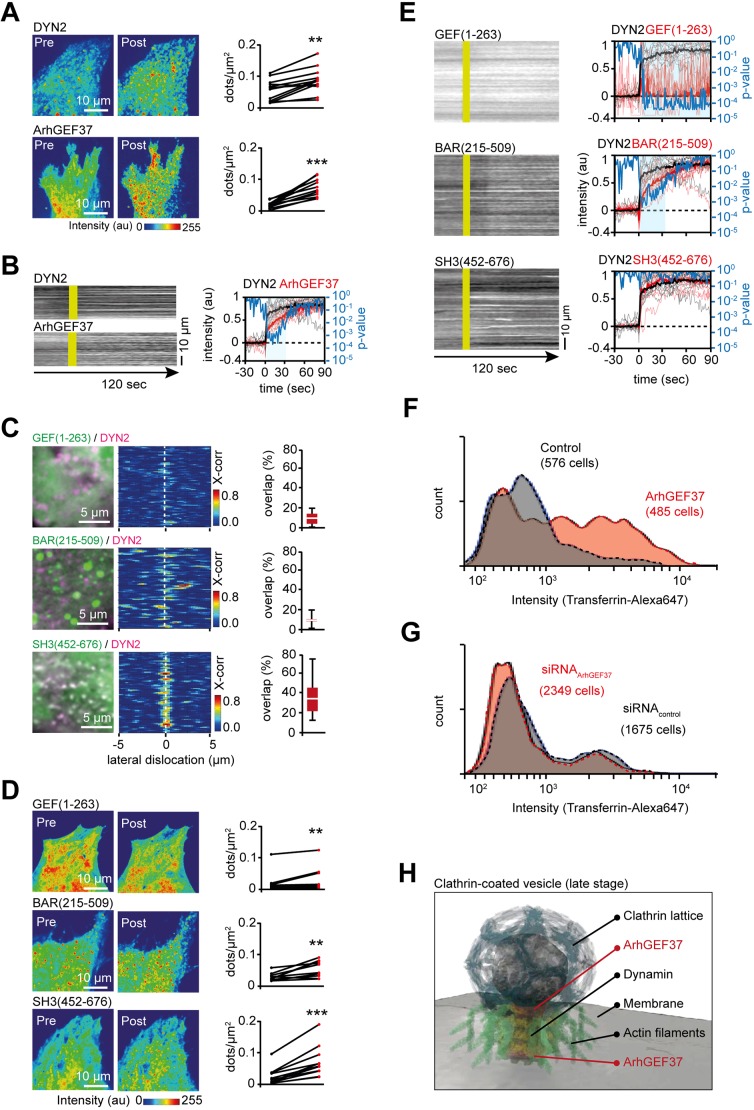
**ArhGEF37 increases endocytosis rate.** (A) Hyperosmotic shock increases DYN2 and ArhGEF37 puncta. Cells expressing DYN2 (top) or ArhGEF37 (bottom) before and after hyperosmotic shock (each construct, *n*=12 cells). (B) DYN2 and ArhGEF37 show differences in recruitment kinetics. Start of hyperosmotic shock is indicated in yellow. To the right, kinetics of ArhGEF37 (red) and DYN2 (black) as well as statistical analysis (blue). Bold lines depict the median, thin lines individual cells. (C) The SH3 domain of ArhGEF37 colocalizes with DYN2. Left: Cells expressing indicated ArhGEF37 constructs (green) and DYN2 (magenta). Middle: Cross-correlation analysis of DYN2 vs ArhGEF37. Right: Overlap percentage for GEF (9±6%, *n*=11 cells), BAR (8±4%, *n*=11 cells) and SH3 (35±6%, *n*=11 cells) domain. Notice, that BAR-domain puncta appear larger than SH3 domain probably because of protein aggregation. (D) Hyperosmotic shock enriches GEF, BAR and SH3 domains at the PM. Quantification of the total number of dots/µm^2^ measured pre (black) and post (red) hyperosmotic treatment (*n*=12, 10, 11 cells for GEF, BAR and SH3 domain, respectively) is shown in the graph to the right of each image. (E) The SH3 but not the BAR domain shows the same kinetics as DYN2. Images on the left show kymographs of hyperosmotic shock (yellow). Graphs on right show the kinetics of individual constructs (red) and DYN2 (black) as well as statistical analysis (blue) (*n*=12, 12, 10, 9 cells for DYN2, GEF; BAR and SH3, respectively). (F) Overexpression of ArhGEF37 increases the rate of endocytosis. FACS analysis 24 h after transfection with ArhGEF37 (red) or control (black). (G) Partial knockdown of ArhGEF37 reduces the rate of endocytosis. Transferrin-Alexa Fluor 647 uptake probed with FACS 48 h post transfection with siRNA_control_ (black) or siRNA_ArhGEF37_ (red). (H) Proposed site of ArhGEF37 action during late phase of CME. A late endocytotic vesicle decorated with clathrin (blue), dynamin (yellow) and actin (green). Based on membrane occupancy and isosurface analysis, ArhGEF37 (red) may enrich at the top and/or base of the stalk, connecting vesicle and membrane. Scale bars: 10 µm (A,B,D,E); 5 µm (C). See also Fig. S4 and Movies 3-6.

To further characterize the interplay between DYN2 and ArhGEF37, we aimed at acutely inducing protein recruitment to the PM. Published work established that hyperosmotic shock decreases membrane tension, thereby reducing turnover of endocytotic proteins (Heuser and Anderson, 1989). In agreement with previous studies (Morlot et al., 2012), hyperosmolarity led to an increase in DYN2 puncta at the PM (Fig. 4A, top and Movie 2). Similarly, a hyperosmotic shock augmented the number of ArhGEF37 puncta at the PM (Fig. 4A, bottom and Movie 3). To rule out that protein accumulation was due to the perturbation protocol (e.g. cell shape changes or focal shift), we expressed cytosolic CFP. We find no changes in CFP intensity, pattern or puncta number upon hyperosmotic shock (Fig. S4A and Movie 4). Finally, we measured recruitment kinetics. As in the cross-correlation experiments, we observed DYN2 recruitment preceding ArhGEF37 enrichment at the PM (Fig. 4B).

To determine the origin of these differences in recruitment kinetics, we tested ArhGEF37 deletion mutants composed of the sole GEF domain (GEF; aa 1-263), the BAR domain (BAR; aa 215-509) or the SH3 domains (SH3; aa 452-676). When expressed in cells, the GEF domain yielded predominantly cytoplasmic localization, whereas BAR and SH3 showed punctate expression pattern at the basal membrane (Fig. 4C, left). As above, we tested for colocalization with DYN2. No significant overlap was found for the GEF or the BAR domain, whereas the SH3 domain showed strong colocalization (overlap percentage 35±16%, *n*=13 cells) (Fig. 4C, right). Likewise, the SH3 domain showed elevated colocalization when coexpressed with CTLA (Fig. S4B).

Next, we applied a hyperosmotic shock to the truncated versions of ArhGEF37. Curiously, we observed for all constructs elevated signal levels at the PM after hyperosmotic shock (Fig. 4D, and Movie 5). To further characterize these unexpected findings, we examined their recruitment kinetics. For the GEF domain, we find an increase in transient puncta, whereas the SH3 domain of ArhGEF37 yielded long-lasting puncta with almost identical kinetics to those of DYN2 (Fig. 4E). Intriguingly, and unlike the SH3 domain, recruitment of the BAR domain strongly resembled full-length ArhGEF37.

Collectively, these studies are consistent with BAR-dependent recruitment of ArhGEF37 to late endocytotic sites, followed by protein–protein interactions through the SH3 domain. As DNMBP, one of the predicted ArhGEF37 homologs (Table S2), has been described to bind to dynamin through its SH3 domain (Salazar et al., 2003), we next performed pull-down assays. Specifically, we used full-length ArhGEF37, the BAR domain and the SH3 domain as bait. We did not detect DYN2 in any pull-down (Fig. S4C). While these experiments do not irrefutably exclude direct interactions, they argue against accessible strong and/or direct contact sites between DYN2 and our constructs.

The experiments to this point indicate that ArhGEF37 is recruited to vesicle scission sites. To elucidate the functional relevance of protein recruitment, we probed in a last set of experiments endocytosis rates upon changes in ArhGEF37 levels. In a first set of experiments, we expressed full-length ArhGEF37 and probed for changes in transferrin uptake. Compared to mock transfection using an empty YFP construct, FACS analysis showed a more than two-fold increase in mean transferrin uptake in the presence of ArhGEF37 (+122%, *n*=3 repeats for each condition, *n*_Control_=1358 cells, *n*_ArhGEF37_=1429 cells after gating) (Fig. 4F). Consistently, a partial knockdown of ArhGEF37 led to a subtle reduction in mean uptake of transferrin compared to that of control knockdown (−11%, *n*=3 repeats for each condition, *n*_Control_=5351 cells, *n*_ArhGEF37_=6952 cells after gating; Fig. 4G, Fig. S4D-H and Table S4), arguing that ArhGEF37 promotes endocytosis.

## DISCUSSION

By taking advantage of structure prediction, biochemical and quantitative image analysis, we identified ArhGEF37 as a regulatory protein involved in CME (Fig. 4H and Movie 6). Kinetics, genetic and chemical perturbation analyses indicate recruitment of ArhGEF37 to late endocytotic vesicles, whereas transferrin uptake experiments (Fig. 4F,G) suggest ArhGEF37 to augment endocytosis rates. However, the precise molecular mechanism of how this is achieved remains elusive.

In the following, we briefly discuss possible implications arising from the experimental findings. ArhGEF37 differs from known BAR-domain proteins in its surface-charge distribution (Fig. 1C,D) and spatio-temporal dynamics (Fig. 2A). It is, thus, plausible to envision that the unique BAR domain mediates positioning during endocytosis. Consistently, full-length ArhGEF37 and the isolated BAR domain show comparable recruitment kinetics (Fig. 4E). Once recruited, our data suggests that ArhGEF37 increases endocytosis rates (Fig. 4F,G) by augmenting the dynamin-dependent vesicle scission rate. Intriguingly, ArhGEF37 carries a RhoGEF domain, raising the possibility that ArhGEF37 can, analogous to other endocytotic proteins (Almeida-Souza et al., 2018; Brinas et al., 2013; Salazar et al., 2003), alter actin-dynamics to augment uptake rates (Fig. 4E and Fig. S4I-L).

However, although recruitment of DYN2 and the SH3 domain of ArhGEF37 coincide spatio-temporally (Fig. 4E), pull-down assays did not identify direct binding between these proteins (Fig. S4C). Hence, our studies do not define any interactions with the canonical endocytic machinery. Finally, when considering the role of DYN2 in clathrin-independent types of endocytosis (Cao et al., 2007; Henley et al., 1998; Sauvonnet et al., 2005), ArhGEF37 might play additional roles beyond CME. Future work will unveil the full scope of functions employed by ArhGEF37 to modulate endocytosis.

## MATERIALS AND METHODS

### Cell culture

HeLa cells (Leibniz Institute DSMZ, ACC-57) and NIH 3T3 embryonic fibroblasts (Leibniz Institute DSMZ, ACC-59) were cultured in Dulbecco's modified Eagle’s medium (DMEM) containing 4.5 g/l D-glucose, GlutaMax-I and Pyruvate (31966-021, Gibco), supplemented with 10% fetal bovine serum (L11-004, BioChrom AG) and 1% penicillin-streptomycin 10 µg/ml (15140-122, Thermo Fisher Scientific). Cells were incubated at 37°C in 5% CO_2_ and passaged two to three times a week.

### Structure prediction and modeling

Prediction of the secondary structure of the individual domains (GEF, BAR, SH3-1 and SH3-2) was performed by PSIPRED (http://bioinf.cs.ucl.ac.uk/psipred). For building structural models of the individual domains, we used homology modeling, a method that uses existing structures of homologous proteins (templates) to build structural models for a given sequence (query). We first performed a search for templates using the fold prediction of pGenTHREADER (http://bioinf.cs.ucl.ac.uk/psipred). The best-fitting structures were then sorted according to the prediction certainty. Top scorers (and the alignments generated by pGenTHREADER) were then chosen for homology modeling (Table S2). By using MODELLERv9.19, 1200 homology models were generated for each individual domain, using a ‘slow’ optimization protocol followed by a ‘slow’ molecular dynamics-based refinement protocol. Next, the generated models of individual domains were ranked based on a normalized DOPE score (similar to a Z-score), with the top scoring model being the one shown in the paper. To generate a model of the BAR dimer structure of ArhGEF37, the modeled individual BAR domains were superimposed on the symmetrical units that form the dimer in the amphiphysin crystal structure (PDB ID: 1URU). The chosen homology models and the modeled BAR dimer were then solvated in a truncated octahedron water box with TIP3P water molecules, in a way that a layer of water of at least 12 Å surrounded the solute in any direction. Na^+^ ions were added to counteract the charges, as well as 150 mM KCl. The solvated systems were then subjected to a 50,000 steps energy minimization, with gradually relaxing constraints, combining steepest descent and conjugated gradient steps. All minimizations were done in explicit solvent, using the AMBER ff14SB force field model and the AMBER 18 software (ambermd.org/). Finally, by using the Adaptive Poisson Boltzmann Software (APBS; http://www.poissonboltzmann.org/), the non-linear Poisson-Boltzmann equation was solved (at a salt concentration of 0.15 M) to obtain the electrostatic potentials of the minimized structures.

### Sequence alignment

Alignments of the BAR domain of ArhGEF37 and DNMBP (Fig. 1B) and the BAR domain of ArhGEF37 with its top scoring homologs (Fig. S1B) were generated using CLUSTALX (http://www.clustal.org/omega/).

### Protein purification

C-terminal His_6_-tagged ArhGEF37 (aa 1-676) was cloned into pET21a vector and transformed into *E.coli* BL21-DE3 cells (70235-3, Millipore). Cells were grown in YT medium containing 16 g/l tryptone/peptone (8952.3, Carl Roth), 10 g/l yeast extract (2363.3, Carl Roth), and 5 g/l NaCl pH 7 at 37°C until an OD_600_ of 0.6–0.7 reached. Protein expression was induced with 0.5 mM IPTG (R0392, Thermo Fisher Scientific) for 24 h at 18°C. Cells were then spun down at 10,000 ***g*** for 30 min at 4°C and the pellet was resuspended in lysis buffer containing 30 mM Tris-HCl (4855.2, Carl Roth), 0.5% Triton X-100 (T9284, Sigma), 45 mM imidazole (3899.2, Carl Roth), 5% glycerol (G6279, Sigma), 1:100 protease inhibitor mix (39106.01, Serva), 1% lysozyme (L4919, Sigma), 0.1% DNAase (D5025, Sigma) pH 7.5 for 15 min on ice followed by sonication. Cell lysates were precipitated by centrifugation at 30,000 ***g*** for 30 min at 4°C. The target protein was purified using immobilized metal-ion affinity chromatography (IMAC) by 3 Hi-Trap-chelating columns (17-0409-03, GE Healthcare) incubated with Ni^2+^ using the Äkta Prime Plus system (11001313, GE Healthcare). Specifically, the protein sample was injected into the system at a flow rate of 5 ml/min and washed with IMAC running buffer containing 20 mM HEPES pH 8, 500 mM NaCl, 45 mM imidazole, 5% glycerol. The target protein was then eluted with 20 mM HEPES pH 8, 500 mM NaCl, 500 mM imidazole, 5% glycerol. The elution fractions were concentrated using Amicon Ultra-4 centrifugal filters 30K (UFC803024, Millipore) and loaded onto a HiLoad 16/600 Superdex 200 pg column (28989335, GE Healthcare) for further purification by using size exclusion chromatography. Finally, all fractions were collected in 20 mM HEPES pH 8, 500 mM NaCl, 5% glycerol and purity was confirmed by SDS/PAGE and western blot. The purified protein was concentrated and stored at −80°C for further studies.

### Mass spectrometry

Sample preparation. Proteins were precipitated by adding three volumes of ice-cold acetone overnight at −20°C. After centrifugation (20,000 ***g*** for 20 min at 4°C) samples were dried and solubilized in 8 M Urea at room temperature (RT). By adding 10 mM (NH_4_)HCO_3_ buffer (pH 7.8) the concentration was reduced to 2 M Urea. Samples were subjected to cysteine reduction and carbamidomethylation using 10 mM Tris (2-carboxyethyl) phosphine at 37°C for 30 min and 30 mM iodoacetamide at RT for 30 min. Protein hydrolyzation was carried out with trypsin (Promega) at a ratio of 1:100 (trypsin:protein) by incubating the samples overnight at 37°C. Tryptic digestion was stopped by adding formic acid to decrease the pH to below 3. Sample desalting was carried out with a tc18 cartridge (Sep-Pak Vac 1cc tc18 cartridge, Waters) according to the manufacturer's instructions. Eluted peptides were dried in a vacuum concentrator and resolved in 0.1% TFA for nano-LC-MS/MS analysis. The final concentration was adjusted to 1 µg/µl.

#### Quality control

Proteolytic digests were checked for complete hydrolyzation after desalting by using monolithic column separation (PepSwift monolithic PS-DVB PL-CAP200-PM, Dionex) on an inert Ultimate 3000 HPLC (Dionex, Germering, Germany) by injection of 1 μg sample. A binary gradient (solvent A: 0.1% TFA, solvent B: 0.08% TFA, 84% ACN) in the range of 5-12% B in 5 min followed by 12-50% B in 15 min at a flow rate of 2.2 μl/min and at 60°C, was applied. UV traces were acquired at 214 nm.

Nano-LC-MS/MS analysis. All samples were analyzed by using an Ultimate 3000 RSLC nano system (Dionex) coupled to a Qexactive HF mass spectrometer (Thermo Fisher Scientific). In total, 1 µg of each sample was injected. Peptides were pre-concentrated on a 75 μm×2 cm C18 trapping column for 10 min using 0.1% TFA (v/v) at a flow rate of 10 μl/min, followed by separation on a 75 μm×50 cm C18 main column (Acclaim Pepmap, Thermo Fisher Scientific) with a 110 min LC gradient in the range of 3-45% B (84% ACN in 0.1% FA) at a flow rate of 250 nl/min. MS survey scans were acquired on the Qexactive mass spectrometer with a 300−1500 mass-to-charge (*m*/*z*) ratio at a resolution of 60,000 using the polysiloxane ion at 371.1012 *m*/z as lock mass. The 15 most-intense ions were subjected to high-energy collision-induced dissociation (HCD), taking into account a dynamic exclusion of 15 s. HCD spectra were acquired with normalized collision energy of 27%. AGC target values were set to 1e6 for MS1 and 5e4 for MS2 scans and maximum injection times were set to 120 ms for MS1 and 50 ms for MS2 scans, respectively. The isolation window was set to 1.6 *m*/z.

Data analysis. The data analysis was performed by using proteome discoverer 1.4, using Mascot 2.6.1 as search and identification engine. The search parameters were set as follows. Protease was selected to be trypsin with full specificity and a maximum of missed cleavage sites of two. The precursor *m*/z tolerance was set to 10 ppm, whereas the fragment *m*/z tolerance was set to 0.02 Da. The searches were performed against a targeted/decoy human UniProt database (downloaded October 26th 2017) containing 40,340 sequences. Carbamidomethylation of cysteine was set as fixed, oxidation of methionine as variable modification. The false discovery rate was set to 1%.

### Quartz crystal microbalance

To create model membranes, 1-palmitoyl-2-oleoyl-sn-glycero-3-phosphocholine (POPC), 1,2-dioleoyl-sn-glycero-3-phosphocholine (DOPC), Cholesterol (Chol), 1,2-dioleoyl-sn-glycero-3 [phosphoinositol-4,5-bisphosphate](triammonium salt) (PI(4,5)P_2_), and lipid brain extract were purchased from Avanti Polar Lipids Inc. (Alabaster, AL). The citrate buffer contained 10 mM trisodiumcitrate (Merck, Darmstadt, Germany) and 150 mM NaCl pH 4.6 (at RT). The HBS buffer consisted of 10 mM HEPES and different NaCl concentration at pH 8.0 (at RT). Solvents (HPLC grade) were purchased either from Merck, Carl Roth or AppliChem (Darmstadt, Germany). For small unilamellar vesicle (SUV) preparation, the respective lipids and cholesterol were dissolved in chloroform/methanol (1:1 v/v) except for PI(4,5)P_2_ that was dissolved in chloroform/methanol/water (20:9:1 v/v/v). The stock solutions were mixed and the organic solvents were removed under a stream of nitrogen above the lipid gel-fluid phase transition temperature. Residual traces of the organic solvent were then evaporated in high vacuum for 4 h at the same temperature and lipid films were stored at 4°C until use. Lipid films (i.e. model membranes) of the following composition (given in molar ratios) were used: POPC/DOPC/Chol/PI(4,5)P_2_ (60:20:10:10), POPC/DOPC/Chol (70:20:10) or the lipid brain extract. Dry lipid films were suspended in the citrate buffer at 60°C for 30 min with subsequent steps of vortex mixing every 5 min. Multilamellar vesicles (MLVs) were extruded 31 times using a polycarbonate membrane with a pore size of 50 nm (Avestin Liposofast, Ottawa, Canada) to obtain SUVs.

#### Preparation of solid supported bilayers

Silicon-coated quartz sensor (QSX 303, 50 nm SiO_2_, 4.95 MHz) were cleaned in 2% (w/v) SDS (SERVA, Heidelberg, Germany) and hydrophilized during a 10-min O_2_-plasma treatment (Harrick Plasma, Ithaca, NY). Subsequently, surfaces were rinsed with ultra-pure water and dried in a stream of nitrogen. To prepare the supported bilayer membrane on the quartz sensor by vesicle rupture, SUVs (0.5 mg/ml, 50 nm) were applied during quartz crystal microbalance with dissipation (QCM-D) measurements. Following membrane formation, the citrate buffer was exchanged to a HBS-Puffer pH 8.0 containing different salt (NaCl) concentrations between 125 mM and 1 M.

#### QCM-D measurements

Quartz crystal microbalance with dissipation (QCM-D) measurements were performed on a Q-Sense E4 QCM-D Analyzer (Q-Sense, Gothenburg, Sweden) equipped with four temperature-controlled flow cells in a parallel configuration. Flow cells were connected to a peristaltic pump (Ismatec IPC, Glattbrugg, Switzerland) employing a flow rate of 80.4 μl/min. Binding analysis was performed at 20°C in HEPES-buffered saline supplemented with NaCl at different concentrations (125 mM to 1 M). Frequency and dissipation shifts of the third overtone resonance frequency of the quartz sensor (QSX 303, 50 nm SiO_2_, 4.95 MHz) were monitored and considered for data evaluation. Calculations were carried out using OriginPro v. 9.1 (OriginLab Corp., Northampton, MA).

### SDS/PAGE and western blots

Brain tissue lysates (PK-AB718-1403-0/7/14, Promo Kine) and other protein samples were diluted 1:1 with 2×Laemmli buffer containing 125 mM Tris-HCl, 0.005% Brilliant Blue (19598.1, Carl Roth), 20% glycerol, 4% SDS (20765.3, Serva) and 5% β-mercaptoethanol (M-7154, Sigma), incubated for 2 min at 92°C and loaded onto 12% polyacrylamide gel (http://www.poissonboltzmann.org/). Primary antibodies used were anti-ArhGEF37 (1:500, HPA043885, Atlas Antibodies), anti-α-tubulin (1:500, 302-211, Synaptic Systems) and anti-HIS_6_ tag (1:500, 18184, Abcam), incubated overnight at 4°C. All secondary antibodies were horseradish peroxidase (HRP)-conjugated (goat-anti rabbit IgG; ADI-SAB-300J, Enzo and anti-mouse IgG, 31430, Invitrogen) used at 1:1000 for 1 h at RT, detected by Biorad clarity western ECL substrate (1705061).

### Transfection

For transient overexpression of ArhGEF37, deletion mutants and other endocytotic proteins, HeLa and NIH 3T3 fibroblast cells were transfected with plasmids 24 h prior to analysis by using FuGENE HD transfection reagent (E2311, Promega) according to described protocol. To achieve optimal knockdown efficiency, HeLa cells were transfected with all three siRNAs together by using Lipofectamine 2000 (11668-019, Invitrogen) according to the manufacturer's protocol and analyzed after 48 h.

### DNA plasmids

Full-length ArhGEF37 (aa 1-676), as well as deletion mutants ArhGEF37 (GEF) (aa 1-263), ArhGEF37 (BAR) (aa 215-509) and ArhGEF37 (SH3) (aa 452-676) were PC-amplified from mouse cDNA (ORF clone MG222459, Origene), and subsequently cloned into Vivid Color-pcDNA 6.2/C-YFP-DEST vector (V357-20, Life Technologies). All plasmids were verified by DNA sequencing before use. The following commercially available mammalian expression vectors were used: FCHO2-pmCherryC1 (Addgene plasmid #27686), CLTA-mCherry (Addgene plasmid #27680), DYN2-pmCherryN1 (Addgene plasmid #27689), FBP17-pmCherryC1 (Addgene plasmid #27688), APPL1-pmCherryC1 (Addgene plasmid #27683) all described by Taylor et al., 2011;DYN2 (K44A)-GFP (Addgene plasmid #22301) from Ochoa et al., 2000, pmCherry Paxillin- (Addgene plasmid #50526) from Kenneth Yamada, Amphiphysin1 (our own, Galic et al., 2012), and cytosol-CFP (our own, Galic et al., 2014). For bacterial expression, C-terminal His_6_-tagged ArhGEF37 (Genebank NM_001001669) was cloned into pET21a vector (ProMab Biotechnologies, Inc., Richmond, CA).

### siRNA knockdown

All siRNAs used in this study were 19-mers including 5′-3′ dTdT sense-strand overhangs custom synthesized by Microsynth (Germany). siRNAs against ArhGEF37 were 5′-AGAUAAGUUGCAAGAUUCA-3′, 5′-ACAGGAGGUAGUUUAGAUA-3′ and 5′-CUGUGAAGUGUGAUACAAA-3′. Scrambled control siRNA used in all experiments was 5′-GGAGAAGAATCCTTAAATT-3′.

### Immunocytochemistry

Cells were fixed in 4% paraformaldehyde (18505, Ted Pella) containing 4% sucrose (S7903, Sigma) in 1×PBS (phosphate-buffered saline, 10010-02, Gibco) for 20 min at RT and subsequently quenched for 20 min with 100 mM NH_4_Cl (K298.2, Carl Roth). Next, cells were permeabilized with 0.1% Triton-X 100 (T9284, Sigma) in PBS containing 2.5% BSA (bovine serum albumin, A9085.25G, Sigma) for 15 min at RT. Cells were then incubated for 1 h at RT with rabbit polyclonal anti-ArhGFE37 (1:800, HPA043885, Atlas Antibody) followed by detection with Alexa-Fluor^®^488 goat anti-rabbit IgG (1:1000, A1108, Life Technologies).

### Fluorescence microscopy

Images were captured using an EMCCD camera (IXON Ultra, DU-888U3-CSO-BV, Andor), 1024×1024 pixel, 13 µm×13 µm pixel size, mounted on the side port of an inverted microscope (Nikon Eclipse Ti). The set-up was equipped with a Yokogawa CSU-X1 spinning-disk scanning unit; 60× or 100× oil objective were used. Lasers for excitation were used at 445 nm (Cobolt, MLD 100 mW), 514 nm (Cobolt, Fandango, 100 mW) and 561 nm (Cobolt, DPL, 100 mW).

### Endocytosis inhibition

Cells were initially cultured in DMEM and then transferred into live-cell imaging solution (LCIS) at equal osmolarity. To inhibit endocytosis, cells were incubated either with 80 µM Dynasore (D7693, Sigma), 30 µM Dyngo-4A (ab120689, Abcam) or 30 µM Pitstop-2 (ab120687, Abcam) for 30 min at 37°C. 0.04% dimethylsulfoxide (DMSO, D2650, Sigma) was used as a vehicle control. For quantification, individual cells were imaged before (pre) and after (post) incubation with drugs. Cells expressing DYN2 (K44A) were fixed 24 h after transfection.

### Hyperosmotic shock

Culture medium (∼300 mOsm) of the cells was exchanged prior to the experiment with ringer buffer containing 120 mM NaCl, 2.5 mM KCl, 2.5 mM CaCl_2_, 1 mM MgCl_2_, 10 mM HEPES, 10 mM glucose (∼300 mOsm). Hyperosmotic shock was induced by addition of 600 mM sucrose, increasing the osmolarity of Ringer’s solution to ∼420 mOsm. For quantification, image acquisition was started 30 s before (pre) hyperosmotic shock and continued for another 90 s (post) hyperosmotic shock.

### Pull-down assay

At a confluency of 60-70%, cells were transfected either with YFP-tagged ArhGEF37, ArhGEF37 (SH3) or ArhGEF37 (BAR). After 24 h of expression, cells were scraped in ice-cold 1×PBS and centrifuged at 500 ***g*** for 5 min. The cell pellet was washed twice with ice-cold 1×PBS, followed by lysis in 200 µl ice-cold Co-IP buffer (10 mM Tris-HCl pH 7.5, 150 mM NaCl, 0.5 mM EDTA and 0.5% NP-40) supplemented with 1 mM serine protease inhibitor (PMSF, Sigma, 329-98-6) and 1:100 protease inhibitor cocktail (Sigma, CO-RO) on ice, with extensive pipetting every 10 min. The cell lysate was centrifuged at 20,000 ***g*** for 10 min at 4°C, supernatant was transferred to a pre-cooled tube and diluted with 300 µl dilution buffer (10 mM Tris-HCl pH 7.5, 150 mM NaCl, 0.5 mM EDTA). For pull down, 25 µl beads (GFP-Trap Magnetic Agarose kit, gtmak-20, Chromotek) were equilibrated in 500 µl ice-cold dilution buffer and washed thrice by using the DynaMag-2 magnet (12321D, Thermo Fisher Scientific). For pull down, previously equilibrated beads were incubated with 450 µl of cell lysates at 4°C for 1 h under rotating conditions. Following, the beads were magnetically separated using DynaMag-2 and washed thrice with ice-cold dilution buffer. At last, beads were suspended in 2× sample buffer for further analysis by SDS/PAGE and western bloting.

### Transferrin uptake

For FACS analysis, transfected HeLa cells were starved for 2 h at 37°C, 5% CO_2_ in pre-warmed serum-free DMEM supplemented with 25 mM HEPES. Cells were kept on ice for 10 min and 20 µg/ml Alexa-Fluor 647 (T23366, Molecular Probes) was added. Cells were incubated for 5 min at 37°C, 5% CO_2_; and further washed once with ice-cold 1×PBS, followed by washing with ice-cold acidic buffer (0.1 M glycine, 150 mM NaCl pH 3) and twice with ice-cold 1×PBS. Cells were dissociated with 6 mM EGTA and centrifuged using a table-top centrifuge at 1000 rpm (100 ***g***) for 3 min at 4°C. Cell pellets were re-suspended in DMEM supplemented with serum and analyzed using FACS (Becton Dickinson, Aria III).

For adherent cells assay, HeLa cells (50-70% confluency) were starved in 300 mOsm Live Cell Imaging Solution (LCIS) containing 140 mM NaCl (9265.2, Carl Roth), 2.5 mM KCl (6781.1, Carl Roth), 1.8 mM CaCl_2_ (CN93.2, Carl Roth), 1 mM MgCl_2_ (KK36.3, Carl Roth), 20 mM HEPES (HN77.4, Carl Roth), 20 mM glucose (6887.1, Carl Roth) and 1% BSA for 30 min at 37°C, 5% CO_2_. Then the cells were kept on ice for 10 min, followed by incubation with 30 µg/ml Alexa-Fluor 647 (T23366, Molecular Probes) for 10 min at 37°C, 5% CO_2_. Cells were washed three times with live-cell imaging solution before fixation with 4% paraformaldehyde for 20 min at RT.

### RNA extraction and qPCR

Total ribonucleic acid (RNA) from cells was extracted using Qiagen RNeasy Mini kit (74104, Qiagen) according to the manufacturer's protocol. First-strand cDNA was synthesized from 1 µg RNA with Thermo Fisher Scientific's SuperScript III Reverse Transcriptase System (18080093) in 20 µl final volume reaction mix containing 30 ng random primers (48190-011, Thermo Fisher Scientific), 1 µl 10 mM dNTPs (18427-013, Thermo Fisher Scientific), 1 µl 0.1 M DTT; 4 µl 5× first-strand buffer, 1 µl RNase OUT (10777-019, Thermo Fisher Scientific) and 1 µl SuperScript III (200 U/µl). Quantitative real-time polymerase chain reaction (qPCR) was performed using a BioRad MyiQ single color real-time PCR detection system with KAPA SYBR FAST Bio-Rad iCycler (KK4606, Sigma) for the real-time amplification detection. Specific primers were designed and tested for qPCR analysis (Table S4).

### Quantification and statistical analysis

#### Image analysis

All images (unless otherwise mentioned) were processed using *Àtrous* wavelet filtering (Olivo-Marin, 2002), and a custom-built MATLAB script (www.mathworks.com) (Fig. S2A,B). For calculation of the overlap percentage, wavelet transformed channels were first binarized and subsequently processed using logical ‘OR’ and ‘AND’ operators, thus resulting in ‘sum’ and ‘overlap’ images, respectively. Finally, the overlap percentage was calculated. For temporal analysis, wavelet transformed stacks were binarized, followed by ‘OR’ operation, therefore generating superimposed images of both channels. Next, an ‘AND’ operation was performed and a mask was generated, representing the ROIs. Finally, an object detection algorithm was used to index island like structures (i.e. ROIs) in the mask. Corresponding to each ROI, mean intensity values were acquired from the original stacks and temporal cross-correlation was calculated (Fig. S2D,E). Software used for image analysis is available on our homepage (https://www.medizin.uni-muenster.de/en/impb/das-institut/nanoscale-forces-in-cells/software/).

#### qPCR analysis

For relative quantification of gene expression, expression of two reference genes (i.e. *OAZ1* and *RPS13*) were measured and used to normalize all qPCR data. Gene expression was calculated with REST software (Pfaffl et al., 2004) according to the following equation:
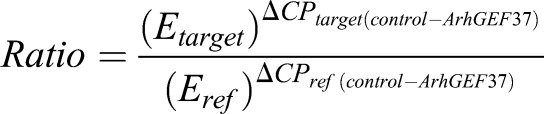
Where *E* is the efficiency, and the target is ArhGEF37; *ref* is *OAZ1* and RPS13, and *CP* is the respective crossing point.

#### Statistics

Experiments are composed of at least three biological repeats, unless stated otherwise in the figure legends. Statistical calculations were accomplished using GraphPad Prism, version 5.03. To test for normal distributions, the Kolmogorov–Smirnov test was applied. To test for equal variance, *F*-test was accomplished. In case of paired experiments, paired *t*-test was performed. For unpaired experiments, the Mann–Whitney test was performed assuming equal variance for comparison. For paired experiments, the Wilcoxon matched-pair signed test was used. Box-plots represent median, interquartile range and whiskers from minimum to maximum. Unless stated in the figure legends, the Mann–Whitney *U*-test was performed for statistics. Following *P* values were used: ns (non-significant), *P*>0.05, *P<0.05, **P<0.01, ***P<0.001.

## Supplementary Material

Supplementary information
