# Supplementary material for: ArhGEF37 assists dynamin 2 during clathrin-mediated endocytosis
Source: J Cell Sci. 2019 May 8;132(9):jcs226530. doi: 10.1242/jcs.226530 (PMC6526708; doi:10.1242/jcs.226530)
Supplement: Supplementary information [file joces-132-226530-s1.pdf]

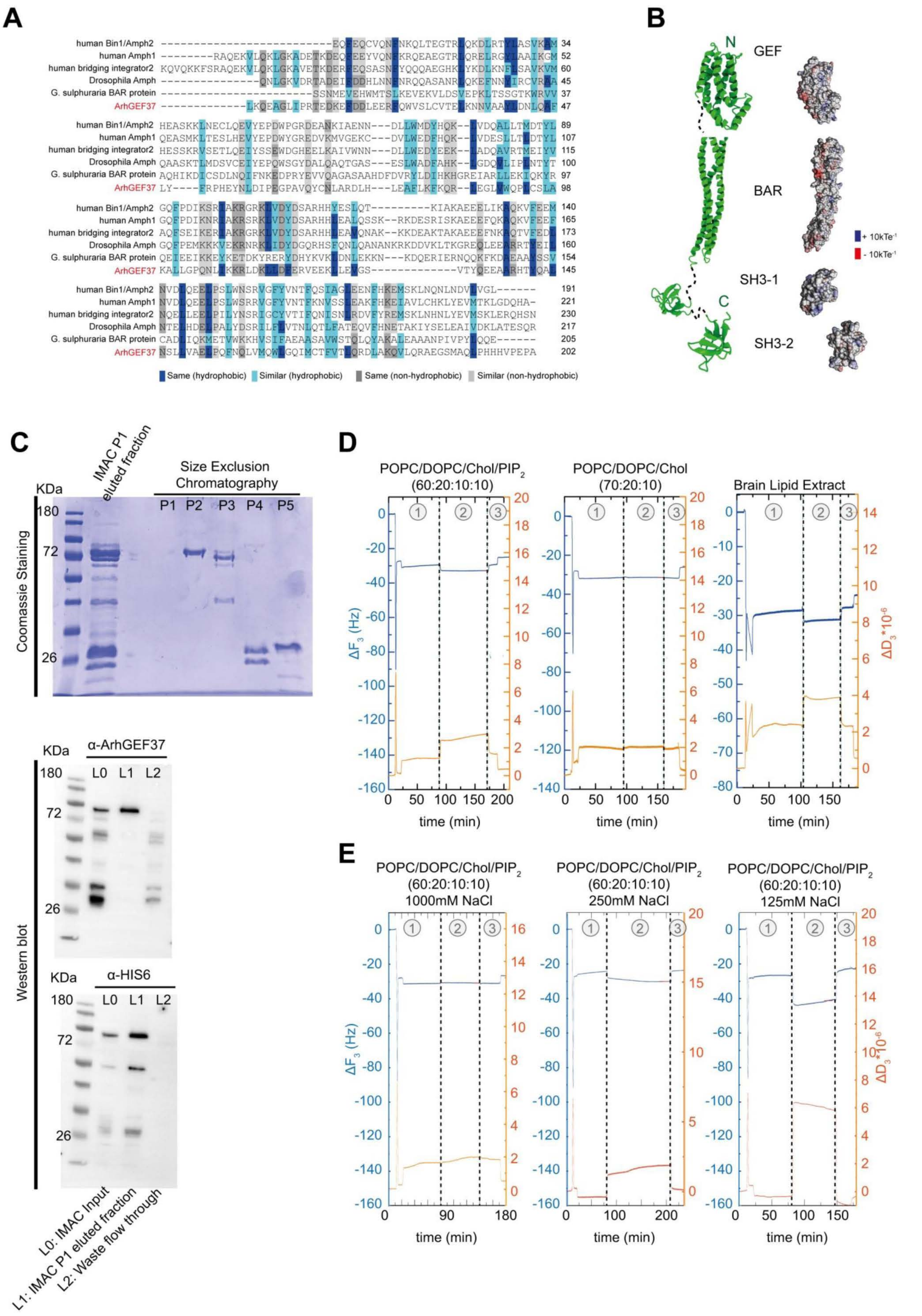

Journal of Cell Science • Supplementary information

Fig. S1. ArhGEF37 structure and membrane binding analysis.

(A) Sequence alignment of BAR domains from ArhGEF37 and different homologs (indicated). Colour-code indicates conserved hydrophobic (blue) and non-hydrophobic (grey) residues. (**B**) Modelled domains of individual ArhGEF37 domains. Corresponding structure models depicting electrostatic surface potential (red, $-10kTe^{-1}$, blue, $+10kTe^{-1}$) are shown next to it. (**C**) Complete Coomassie and Western blots (corresponding to **Fig. 1E**). (**D**) From left to right, measurements of ArhGEF37 binding to POPS/DOPC/Chol/PI(4,5)P_2, POPC/DOPC/Chol and Brain lipid extract (corresponding to **Fig. 1E**). (**E**) From left to right, measurements of ArhGEF37 binding to POPS/DOPC/Chol/PI(4,5)P_2 at 1000 mM, 250 mM and 125 mM, respectively (corresponding to **Fig. 1E**).

Journal of Cell Science • Supplementary information

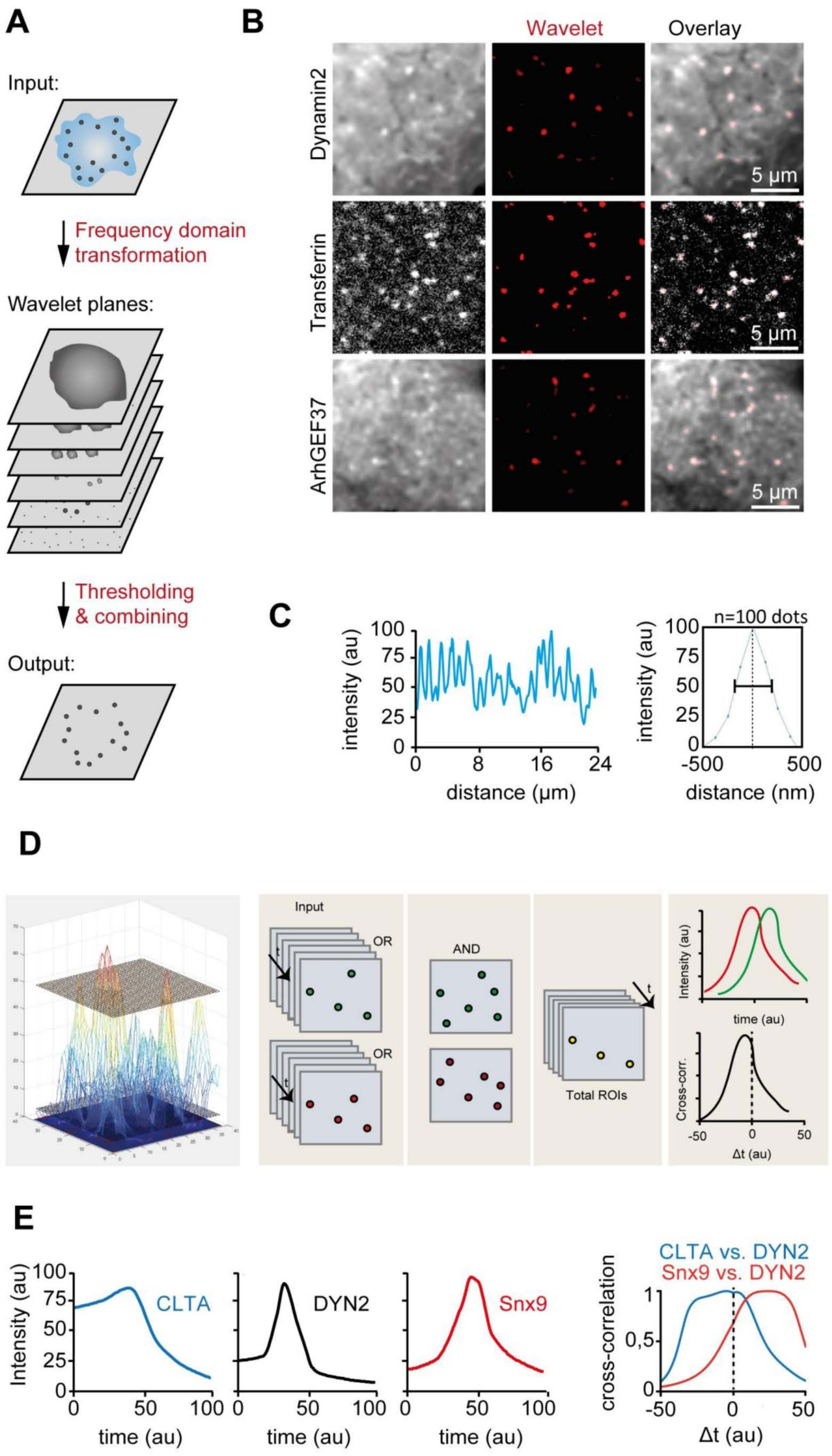

Journal of Cell Science • Supplementary information

Fig. S2. Image analysis tools to study ArhGEF37 function. (**A**) Scheme depicting the principle of *à trous* wavelet filtering. In brief, the filter divides the input image into wavelet planes based on different frequencies of signals in the image. The 1st order wavelet plane consists of noise (highest frequency), followed by wavelet planes with decreasing frequencies associated with increasing structure size in the spatial domain. By combining the 2nd and 3rd wavelet planes, followed by a subsequently thresholding, the final output image is generated. (**B**) Quality controls for *à trous* wavelet filtering analysis. To the left, raw images of cells transfected with fluorescently labelled Dynamin2 (DYN2), transferrin and ArhGEF37, respectively. In the middle, result of *à trous* wavelet filtering for respective images (red). To the right, overlay of raw data (grey) and filter results (red). (**C**) Line-scan (left) and overlay of 100 individual puncta (right) suggest diffraction-limited ArhGEF37 aggregates. (**D**) Scheme showing workflow of custom made script for temporal cross-correlation analysis. In brief, individual puncta were isolated, and intensity profiles measured along the z-axis (i.e. time). To determine the temporal order of appearance, intensity profiles within individual puncta (green and red) are then cross-correlated. (**E**) *In silico* testing of temporal cross-correlation analysis. Published data on recruitment kinetics of CTLA, DYN2 and Snx9 was used to generate intensity traces. Note that the software accurately predicts that CTLA precedes DYN2 (blue) while Snx9 follows DYN2 (red). Scale bar, (B) 5 µm.

Journal of Cell Science • Supplementary information

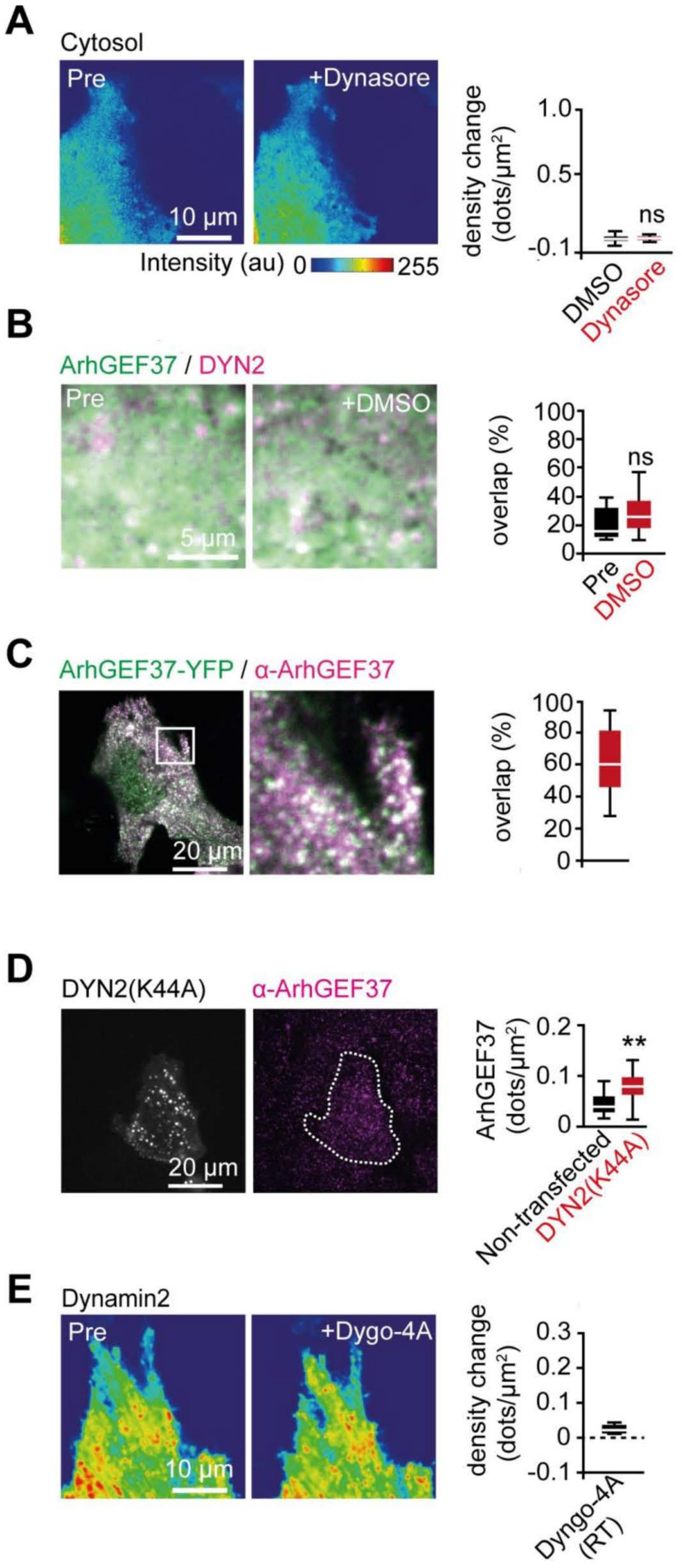

Journal of Cell Science • Supplementary information

Fig. S3. Perturbation controls for ArhGEF37 recruitment. (**A**) No pattern formation in cytosol after Dynasore addition. To the left, cell expressing cytosolic CFP before (pre) and after (post) exposure to Dynasore. To the right, analysis for change in dots/μm^2 upon addition of DMSO (0.00±0.03 dots/μm^2, black, n = 12 cells, mean±SD) and Dynasore (0.00±0.01 dots/μm^2, red, n = 13 cells, mean ± SD). (**B**) DMSO does not trigger increased co-localization of DYN2 and ArhGEF37. Cells were transfected with ArhGEF37 (green) and DYN2 (magenta). Note no significant change in total overlap percentage upon DMSO addition (pre: 21 ± 10%, black; post: 27 ± 13%, red; n = 11 cells, mean ± SD). (**C**) Specificity test for ArhGEF37 antibody. Cells were transfected with ArhGEF37 for 24 hours, fixed and stained with antibody directed against ArhGEF37. Following wavelet-transformation, overlap percentage was determined (62±19%, n = 19). Note: since antibody directed against ArhGEF37 yields multiple bands of various molecular weights on Western blot (**Supplemental Fig. 4G**), unspecific binding partners need to be considered. (**D**) Expression of dominant-negative DYN2(K44A) yields an increase in ArhGEF37 antibody signal. Cells transfected with DYN2(K44A) for 24 hours were fixed and stained with antibody directed against ArhGEF37. Following wavelet-transformation, puncta density was determined for transfected (0.08±0.02 dots/μm^2; n = 35 cells) and non-transfected (0.04±0.02 dots/μm^2, n = 18 cells) cells. (**E**) Temperature shift changes Dyngo4A-dependent DYN2 recruitment. Cells were transfected with DYN2 for 24 hours and incubated for 30 minutes with Dyngo-4A at room temperature. Note differences in enrichment of DYN2 to the membrane compared to 37 °C (shown in **Fig. 3A**). Scale bars, (A, E) 10 μm; (B) 5 μm; (C, D) 20 μm.

Journal of Cell Science • Supplementary information

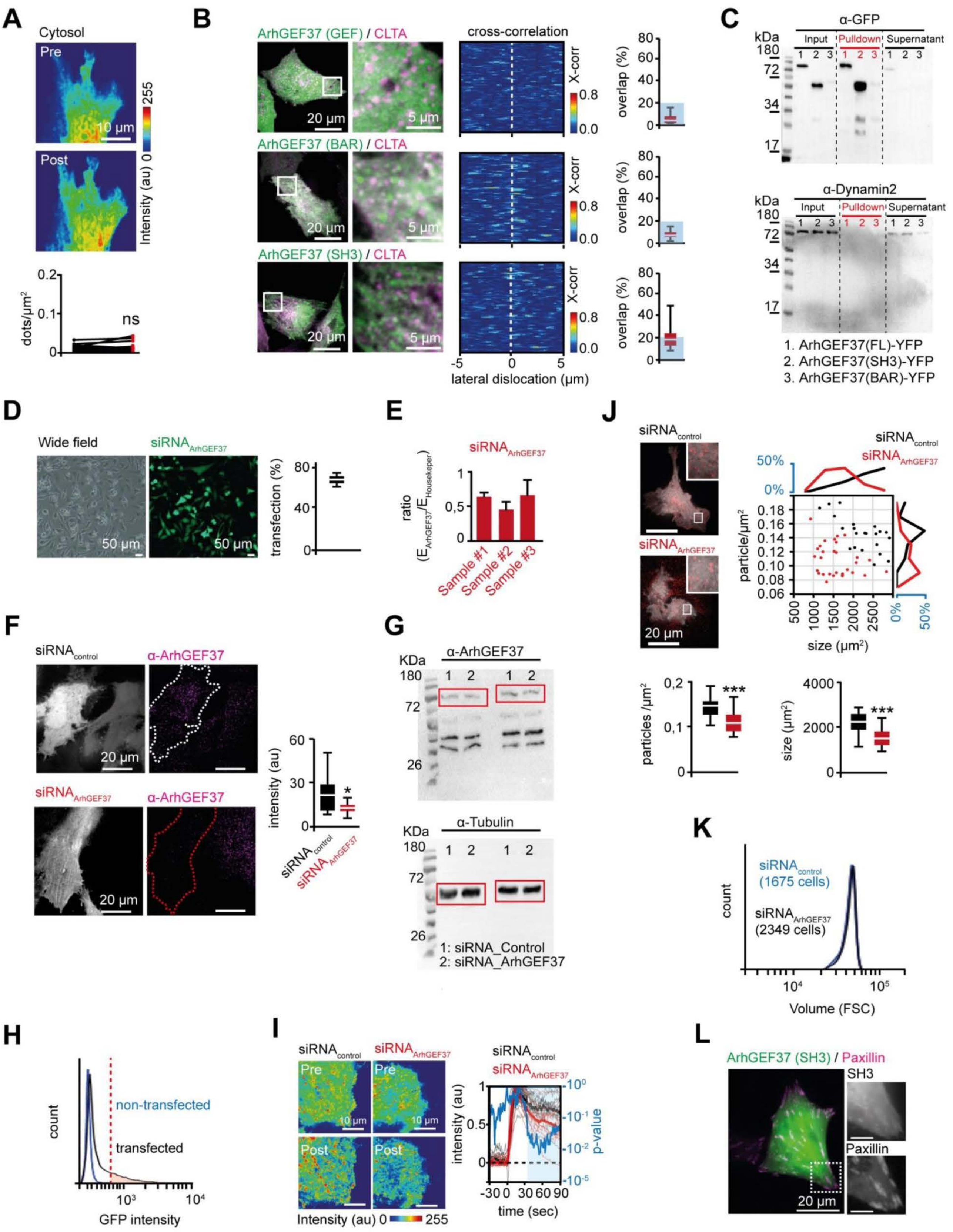

Journal of Cell Science • Supplementary information

Fig. S4. Localization controls and ArhGEF37 knockdown validation. (**A**) Hyperosmotic shock does not change cytosolic signal. Cells expressing cytosolic CFP before (pre) and after (post) hyperosmotic shock. Below, quantification of particles (dots/µm^2) is shown (n = 11 cells). (**B**) Co-localization for different ArhGEF37 domains and CLTA. Left, cells co-expressing indicated ArhGEF37 constructs (green) and CLTA (magenta). Middle panel depict spatial cross-correlation analysis of truncated versions of ArhGEF37 vs. CLTA. To the right, overlap percentage for CTLA and the GEF (5 ± 4%, n = 21 cells), BAR (7 ± 3%, n = 16 cells) and SH3 (20 ± 9%, n = 18 cells) domains (Mean ± SD). Blue boxes in the graphs are added as guidance to the eye. (**C**) Pulldown assay yields no apparent interaction between ArhGEF37 and Dynamin2. From left to right, cell lysate (left), pulldown (middle) and supernatant (right) of cells transfected with full length ArhGEF37 (lane 1), the isolated SH3 domain of ArhGEF37 (lane 2), and the isolated BAR domain of ArhGEF37 (lane 3). Upon pulldown, samples were loaded on gel and stained with antibodies directed against GFP (top gel) and Dynamin2 (bottom gel), respectively. (**D**) Representative image of cells co-transfected with siRNA directed against ArhGEF37 (siRNA$_{ArhGEF37}$) and fluorescence marker (green). Graph depicts transfection efficiency (65 ± 3% n = 4 technical repeats, mean ± SD). (**E**) Analysis of ArhGEF37 knockdown efficiency via qPCR. 48 hours post transfection, cells transfected with siRNA$_{ArhGEF37}$ yield 31± 9% (S1), 53± 23% (S2) and 26± 40% (S3) reduction in mRNA levels (3 biological repeats, each with n = 3 technical repeats, median ± SD). Note that transfection efficiency of 65 ± 3% needs to be considered. (**F**) Knockdown of ArhGEF37 yields reduced ArhGEF37 immunofluorescence signal. To the left cells transfected with siRNA$_{control}$ (black, top) or siRNA$_{ArhGEF37}$ (red, bottom) are shown. Difference in immunofluorescence in control (21 ± 11 au, black, n = 17

Journal of Cell Science • Supplementary information

cells) and ArhGEF37 knockdown (12 ± 3 au, red, n = 19 cells) are shown to the right. (Mean ± SD; Mann Whitney test; * $p \leq 0.05$). (**G**) Western blot analysis of ArhGEF37 knockdown efficiency. Cells were transfected for 48 hours with $siRNA_{control}$ or $siRNA_{ArhGEF37}$. Upon isolation, protein samples were loaded and stained with antibody directed against ArhGEF37 (top) and tubulin (bottom). Two separate biological repeats yield signal reduction of 38% (left) and 22% (right), respectively. Again, note that transfection efficiency of 65±3% needs to be considered. (**H**) FACS analysis (10'000 cells/condition) depicting signal separation for GFP-transfected cells (black) vs. non-transfected cells (blue). Threshold used in **Fig. 4G** for gating (dashed red line) was set at an arbitrary grey value of 670, yielding <1% false positives. (**I**) ArhGEF37 retains DYN2 at endocytotic sites. Cells co-transfected with $siRNA_{control}$ (black) or $siRNA_{ArhGEF37}$ (red) and fluorescently tagged DYN2 before (top) and after (bottom) hyperosmotic shock. To the right, kinetics and statistical analysis (blue) are shown. As above, bold lines depict the median, thin lines individual experiments (n = 14 cells for $siRNA_{control}$ and $siRNA_{ArhGEF37}$, respectively). (**J**) Knockdown of ArhGEF37 reduces transferrin uptake and area of adherent cells. Cells transfected with fluorescence marker and control siRNA or siRNA directed against ArhGEF37 after incubation with transferrin-Alexa647 for 10 minutes at 37 °C. To the right, scatter plot depicting cell area vs. transferrin uptake for $siRNA_{control}$ (black, n = 22 cells) and $siRNA_{ArhGEF37}$ (red, cells, n = 29 cells), respectively. Below, quantification of transferrin uptake for $siRNA_{control}$ (0.14±0.02 particles/μm^2, black) and $siRNA_{ArhGEF37}$ (0.10±0.02 particles/μm^2, red), respectively, show a slight but significant reduction in transferrin uptake. Likewise, quantification of cell area upon transfection with $siRNA_{control}$ (2216±490 μm^2, black) and $siRNA_{ArhGEF37}$ (1585±446 μm^2, red) show a

Journal of Cell Science • Supplementary information

significant difference in cell area. (**K**) Knockdown of ArhGEF37 does not change cell volume in FACS analysis. Cells co-transfected with fluorescence marker and $siRNA_{control}$ (blue) or $siRNA_{ArhGEF37}$ (black). Forward scatter does not yield apparent changes in cell volume. (**L**) The SH3 domain of ArhGEF37 localizes to paxillin-positive focal adhesions. Cells were co-transfected with the isolated SH3 domain of ArhGEF37 (green) and the focal adhesion protein paxillin (magenta). Note slight enrichment of the SH3 domain of ArhGEF37 at focal adhesions. Statistics: * $p \leq 0.05$, ** $p \leq 0.01$, *** $p \leq 0.001$; Mann Whitney test; Error bars represent SEM. Scale bars, (A, I) 10 µm; (B, L) 20 µm, 5 µm; (D) 50 µm; (F) 20 µm.

Journal of Cell Science • Supplementary information

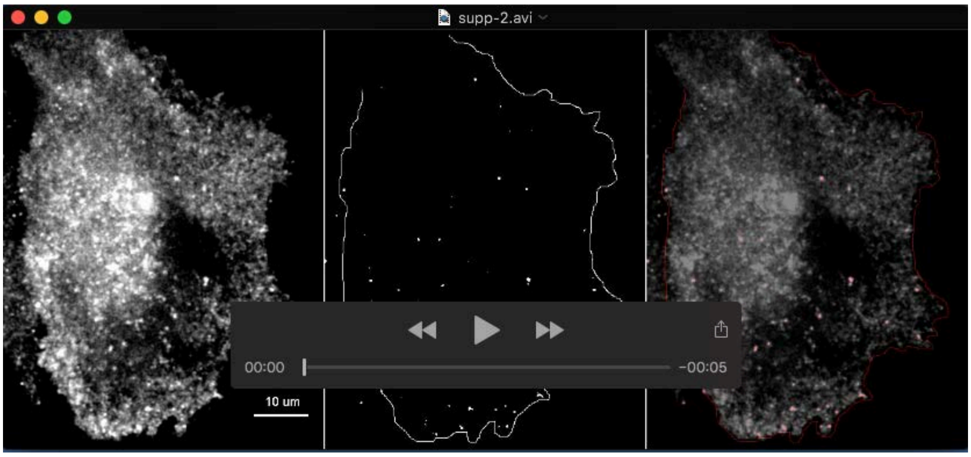

Movie 1. ArhGEF37 recruitment analysed via *à trous* wavelet filtering. HeLa cell transfected with ArhGEF37. To the left, raw data of basal PM. In the middle, same movie subjected to *à trous* filtering. To the right, overlay of raw (grey) and filtered (red) data. Frames captured at 1 Hz. Scale bar, 10 µm.

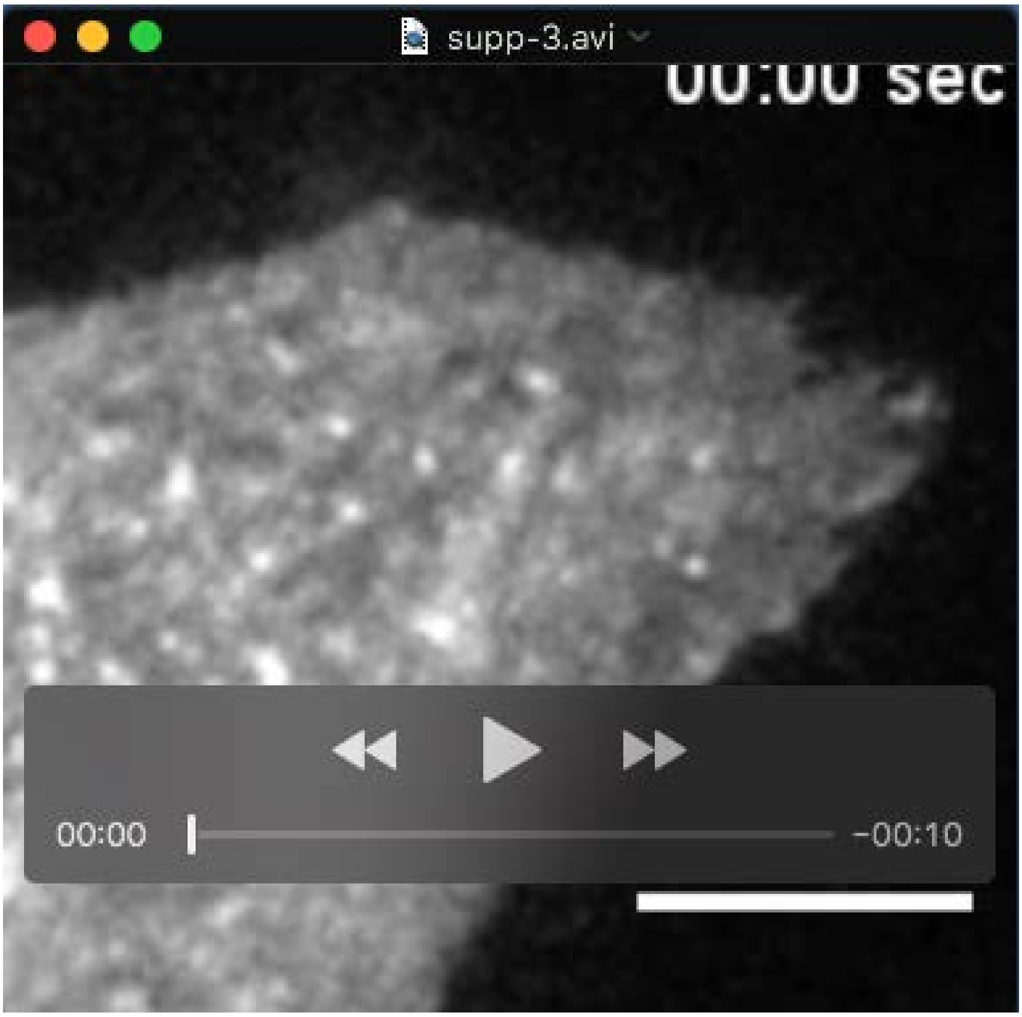

Movie 2. DYN2 recruitment to the PM increases upon hyperosmotic shock. HeLa cell transfected with DYN2 and subjected to hyperosmotic shock (star). Individual frames captured at 1 Hz. Scale bar, 10 µm.

Journal of Cell Science • Supplementary information

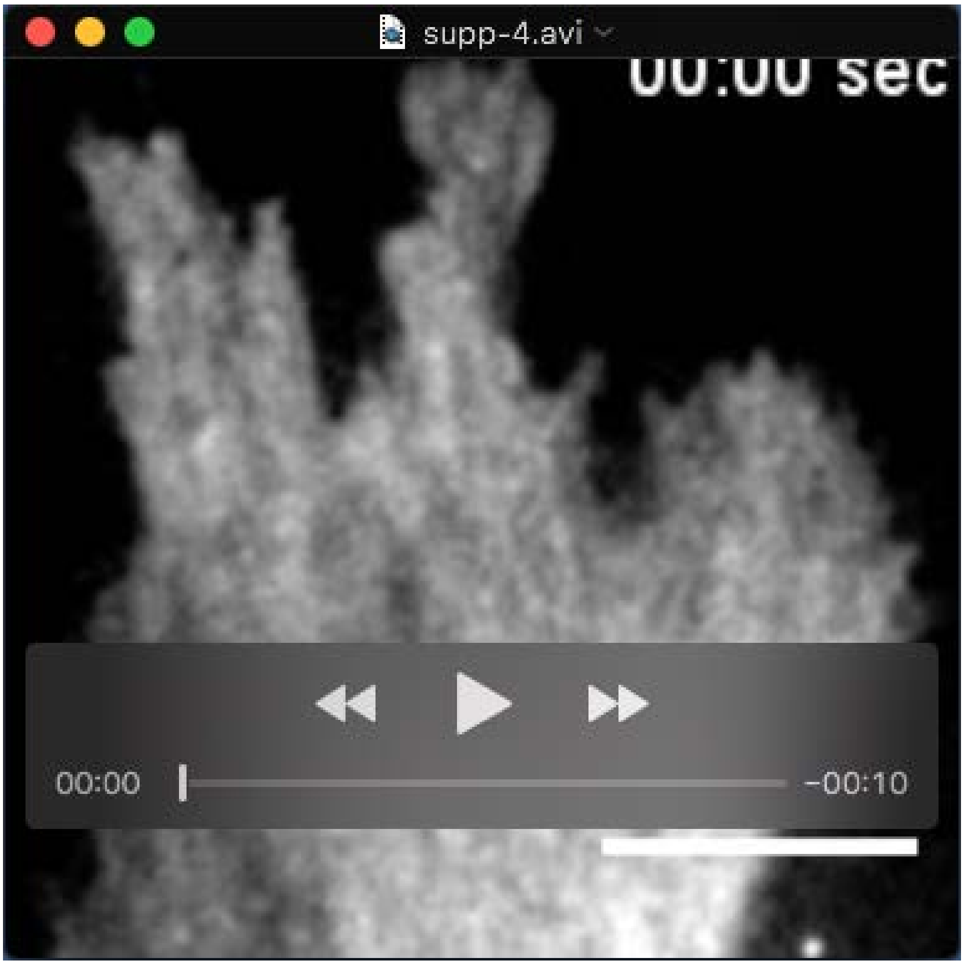

Movie 3. ArhGEF37 recruitment to the PM increases upon hyperosmotic shock. HeLa cell transfected with ArhGEF37, followed by hyperosmotic shock (star). Frames were taken at 1 Hz. Scale bar, 10 µm.

Journal of Cell Science • Supplementary information

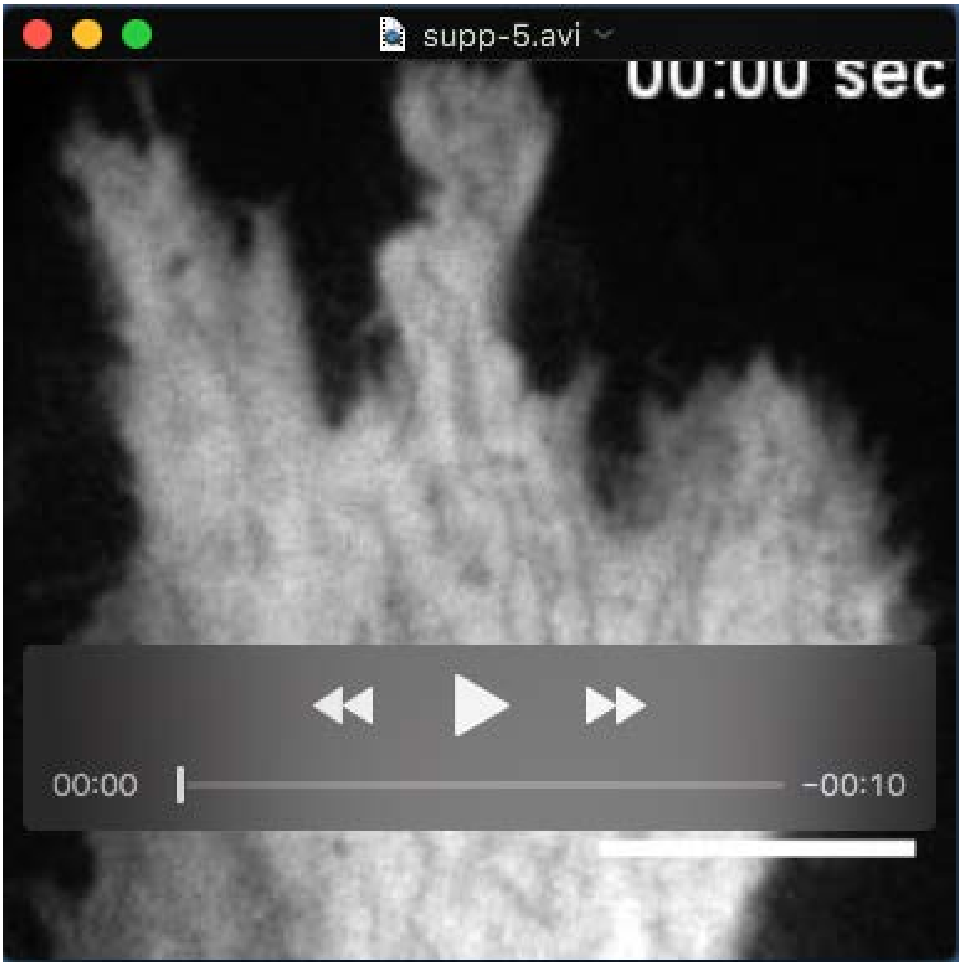

Movie 4. No change in cytosolic perturbation after hyperosmotic shock. HeLa cell transfected with cytosolic marker and subjected to hyperosmotic shock (star). Individual frames were taken at 1 Hz. Scale bar, 10 µm.

Journal of Cell Science • Supplementary information

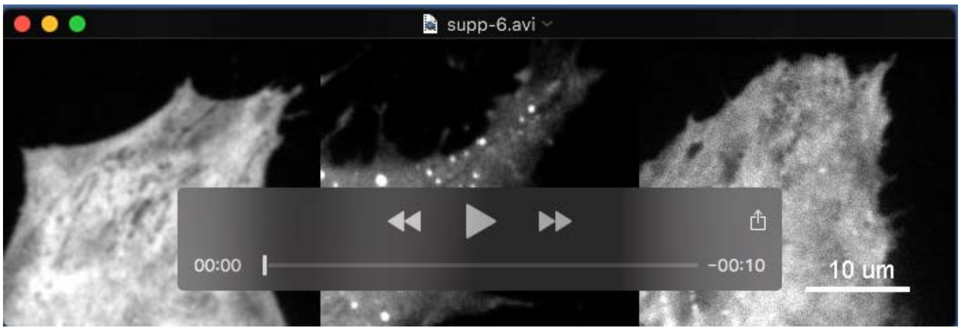

Movie 5. PM recruitment of BAR and SH3 domain of ArhGEF37 increase after hyper-osmotic shock. HeLa cells transfected with truncated versions of ArhGEF37 (GEF; BAR, SH3), and subjected to hyperosmotic shock (star). Individual frames were taken at 1 Hz. Scale bar, 10 µm.

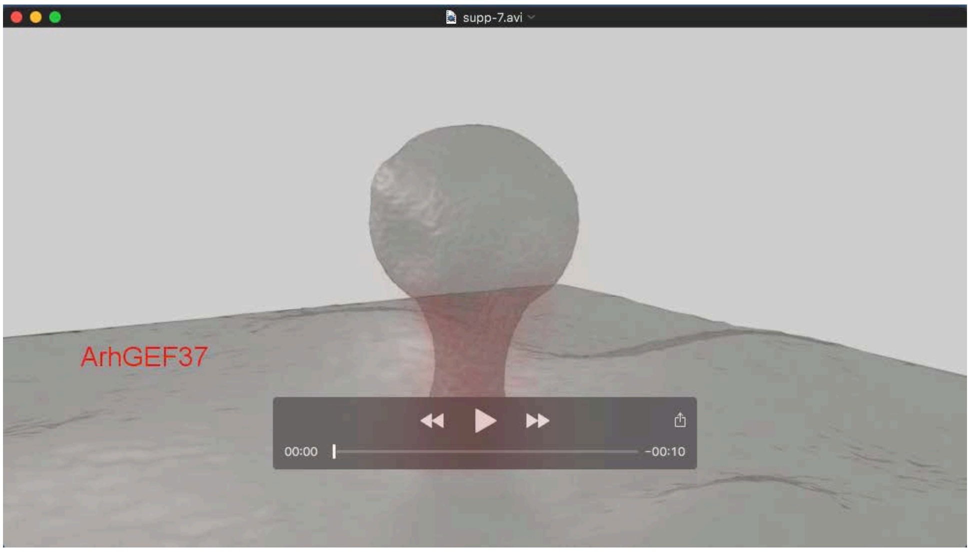

Movie 6. 3D model depicting potential sites for ArhGEF37 enrichment during late phase of CME. Clathrin (blue), filamentous actin (green), Dynamin (yellow), and ArhGEF37 (red) are shown.

Journal of Cell Science • Supplementary information

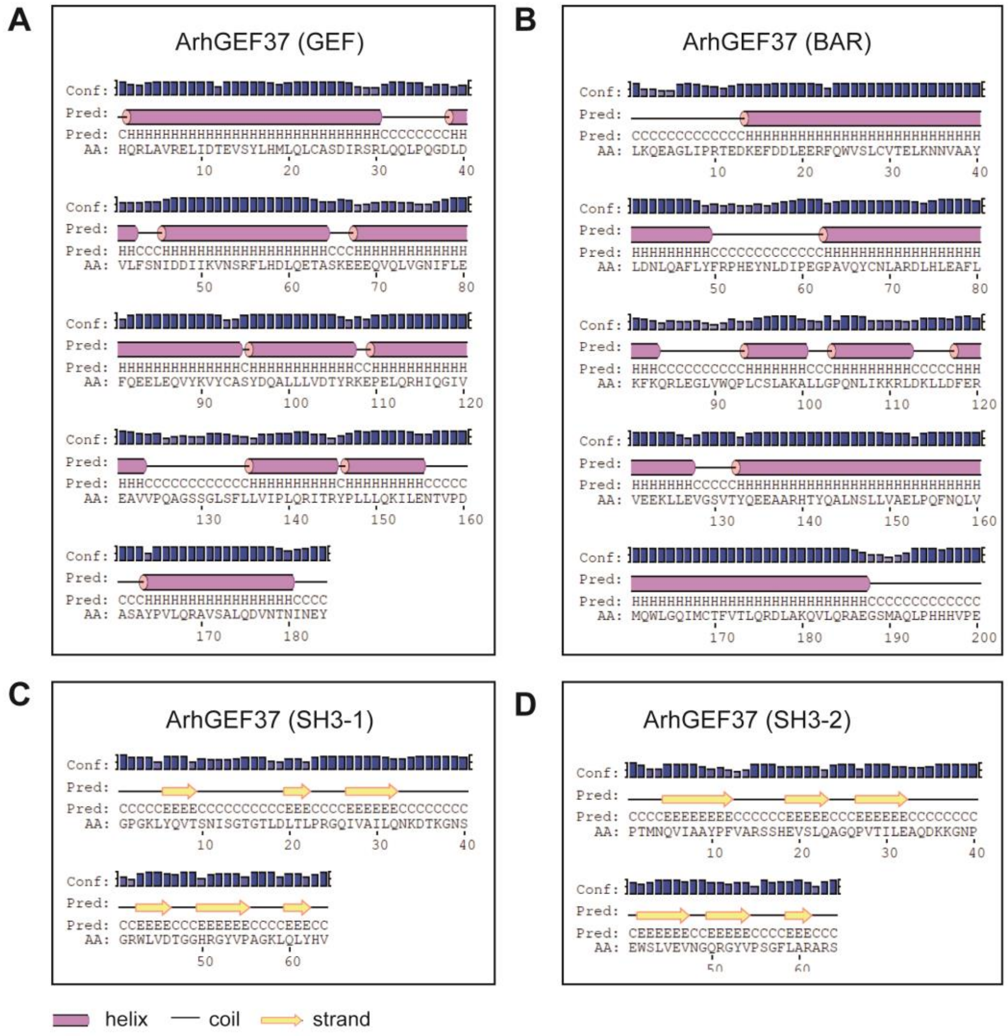

Table S1. Protein secondary structure prediction. Graphical representation of secondary structures predicted via PSIPRED for the following ArhGEF37 domains: GEF domain (**A**), BAR domain (**B**) and the two SH3 domains (**C** and **D).** For every amino acid, blue bars indicate confidence of the prediction. Black lines (letter code C), yellow arrows (letter code E) or purple cylinders (letter code H) indicate a predicted coil region, β-strand or α-helix, respectively.

Domain	PDB ID	Homologs
GEF	3EO2	RhoGEF domain of h-neuroepithelial cell transforming gene-1
	3GF9	Crystal structure of Human intersectin 2 RhoGEF domian
	1F5X	NMR structure of the Y174 autoinhibited DBL homology domain
BAR	4AVM	Crystal structure of N-BAR domain of h-bridging integrator 2
	2FIC	Crystal structure of BAR domain from human Bin1/Amphiphysin II
	1URU	Amphiphysin BAR domain from *Drosophila*
	4ATM	Crystal structure of BAR domain of human Amphiphysin, isoform 1
	3CAZ	Crystal structure of a BAR protein from Galdieria sulphuraria
SH3-1	1UHC	Solution structure of RSG1 RUH-002, SH3 domain of human DNMBP/ K1AA1010 protein
	1UG1	SH3 domain of hypothetical protein BAA76854.1
	1SEM	Structural determinants of peptide-binding orientation & of sequence specificity in SH3 domains.
SH3-2	1UHC	Solution structure of RSG1 RUH-002, SH3 domain of human DNMBP/ K1AA1010 protein
	2KBT	Attachment of an NMR-invisible solubility enhancement tag
	1OOT	SH3 domain from a *S. cerevisiae* hypothetical 40.4 kDa protein
	2K9G	Solution structure of 3rd SH3 domain of the Cin85 adapter protein

Table S2. Structures used for homology modeling. For each domain, both the PDB code and the name of the used homologs are indicated.

Journal of Cell Science • Supplementary information

ArhGEF37 (GEF)

Conf.	Net Score	P-value	Homology
CERT	93.853	2e-08	3eo2
CERT	91.089	3e-08	3gf9
CERT	90.799	3e-08	2dfk
CERT	88.703	6e-08	1f5x
CERT	84.763	1e-07	1kz7
CERT	83.877	2e-07	1foe
CERT	82.708	2e-07	2pz1
CERT	81.786	3e-07	2vrw
CERT	81.746	3e-07	1ki1
CERT	81.581	3e-07	3jv3
CERT	80.679	4e-07	3p6a
CERT	80.094	4e-07	4gyv
CERT	80.028	4e-07	2z0q
CERT	79.170	5e-07	2rgn
CERT	78.270	6e-07	2kr9
CERT	78.029	7e-07	1txd
CERT	77.788	7e-07	1xcg
CERT	75.961	1e-06	3ky9
CERT	75.921	1e-06	1nty
CERT	74.619	1e-06	4gou
CERT	73.090	2e-06	1by1
CERT	72.855	2e-06	4gzu
CERT	58.196	7e-05	3mpx
HIGH	53.427	2e-04	1dbh
HIGH	51.539	3e-04	3ksy
LOW	32.702	0.025	3i9y
LOW	32.144	0.029	4m7c
LOW	31.665	0.032	3qou
LOW	31.659	0.032	3edv
LOW	31.304	0.035	3pdy

ArhGEF37 (BAR)

Conf.	Net Score	P-value	Homology
CERT	80.653	4e-07	4avm
CERT	80.423	4e-07	2fic
CERT	77.736	7e-07	1uru
CERT	77.724	7e-07	4atm
CERT	66.051	1e-05	3caz
HIGH	54.144	2e-04	2d4c
HIGH	51.949	3e-04	1zww
HIGH	50.475	4e-04	1i4d
HIGH	49.456	5e-04	4akv
MEDIUM	45.234	0.001	2raj
MEDIUM	44.748	0.002	4nsw
MEDIUM	43.403	0.002	2z0v
MEDIUM	41.858	0.003	3fhn
MEDIUM	39.811	0.005	2q12
MEDIUM	39.464	0.005	1u5p
MEDIUM	39.176	0.006	3i2w
MEDIUM	39.102	0.006	2q13
MEDIUM	38.658	0.006	4h8s
MEDIUM	38.172	0.007	3g9g
MEDIUM	37.954	0.007	3pdy
MEDIUM	37.818	0.008	2j69
MEDIUM	37.668	0.008	2v0o
MEDIUM	37.373	0.009	3zkv
MEDIUM	37.007	0.009	3dyt
LOW	36.647	0.010	1u4q
LOW	36.039	0.012	4jeh
LOW	36.030	0.012	1hci
LOW	36.002	0.012	4fzs
LOW	35.575	0.013	2r17
LOW	35.470	0.013	2fji

ArhGEF37 (SH3-1)

Conf.	Net Score	P-value	Homology
MEDIUM	46.009	0.001	1ug1
MEDIUM	43.404	0.002	1uhc
MEDIUM	43.403	0.002	1sem
MEDIUM	42.831	0.002	1gri
MEDIUM	42.625	0.003	2drm
MEDIUM	42.326	0.003	1oot
MEDIUM	41.910	0.003	2da9
MEDIUM	41.396	0.003	2kbt
MEDIUM	40.912	0.004	1x2k
MEDIUM	40.793	0.004	2rf0
MEDIUM	40.763	0.004	2k9g
MEDIUM	40.645	0.004	2ydl
MEDIUM	40.511	0.004	2dl4
MEDIUM	40.483	0.004	3ulr
MEDIUM	40.384	0.004	2jte
MEDIUM	40.367	0.004	3ua7
MEDIUM	40.354	0.004	4hvw
MEDIUM	40.312	0.004	3nmz
MEDIUM	40.058	0.005	2d1x
MEDIUM	40.048	0.005	2vwf
MEDIUM	39.937	0.005	2yun
MEDIUM	39.892	0.005	3zl7
MEDIUM	39.883	0.005	2cre
MEDIUM	39.797	0.005	2dl7
MEDIUM	39.630	0.005	2js2
MEDIUM	39.565	0.005	2a08
MEDIUM	39.490	0.005	1zsg
MEDIUM	39.373	0.005	2ak5
MEDIUM	39.343	0.005	1uti
MEDIUM	39.313	0.005	1ng2

ArhGEF37 (SH3-2)

Conf.	Net Score	P-value	Homology
HIGH	54.277	2e-04	1uhc
HIGH	47.418	8e-04	2kbt
HIGH	46.865	9e-04	1oot
HIGH	46.802	1e-03	2k9g
MEDIUM	46.559	0.001	2ydl
MEDIUM	46.474	0.001	1gri
MEDIUM	46.304	0.001	2dl7
MEDIUM	45.693	0.001	1sem
MEDIUM	45.324	0.001	2jte
MEDIUM	44.805	0.002	1zlm
MEDIUM	44.546	0.002	2yun
MEDIUM	44.508	0.002	1uti
MEDIUM	44.454	0.002	1zsg
MEDIUM	44.423	0.002	2g6f
MEDIUM	44.353	0.002	3m0u
MEDIUM	44.344	0.002	2a08
MEDIUM	44.335	0.002	2d8h
MEDIUM	44.229	0.002	1u5s
MEDIUM	44.115	0.002	1x2k
MEDIUM	44.077	0.002	3ua7
MEDIUM	44.002	0.002	4ag1
MEDIUM	43.919	0.002	1neg
MEDIUM	43.829	0.002	3ngp
MEDIUM	43.745	0.002	2drm
MEDIUM	43.679	0.002	1wyx
MEDIUM	43.667	0.002	1x69
MEDIUM	43.578	0.002	2xmf
MEDIUM	43.570	0.002	4afq
MEDIUM	43.515	0.002	3ulr
MEDIUM	43.475	0.002	2ak5

Conf. : The hit confidence category

Net Score: The GenTHREADER raw score

Table S3. Fold-based homolog search. A list of homologs found using the fold-based homolog searching server pGenTHREADER. For every individual domain, the 30 top scoring hits are detailed with indications of the level of confidence, the pGenTHREADER Net Score, the p-value and the PDB accession code.

Journal of Cell Science • Supplementary information

Gene	RefSeq access no.	Forward primer (5´-3´)	Reverse primer (5´-3´)
ArhGEF37	NM_001001669	ATCCTCCAGGTCAGGGAGT	TGCGGCAACTGCTGGAG
		TGAACCAGGTCATAGCCGC	CCAAGAAGCCAGAAGGCACA
OAZ	NM_004152	TAACTGGCCAACAGTGCTGA	ATGAAGACATGGTCGGCTCG
RPS13	NM_001017	GCTCTCCTTTCGTTGCCTGA	TAGGGTAAAGCCGATGGGA

Table S4. List of forward and reverse primers used for qPCR. OAZ stands for Ornithine decarboxylase antizyme, and RPS13 for Ribosomal protein S13.

Journal of Cell Science • Supplementary information
